# CPSF6 promotes HIV-1 preintegration complex function

**DOI:** 10.1128/jvi.00490-25

**Published:** 2025-04-09

**Authors:** Evan Chaudhuri, Sooin Jang, Rajasree Chakraborty, Rajalingam Radhakrishnan, Bjarki Arnarson, Prem Prakash, Daphne Cornish, Nicholas Rohlfes, Parmit K. Singh, Jiong Shi, Christopher Aiken, Edward Campbell, Judd Hultquist, Muthukumar Balsubramaniam, Alan N. Engelman, Chandravanu Dash

**Affiliations:** 1 Center for AIDS Health Disparities Research, Nashville, Tennessee, USA; 2 Department of Biochemistry, Cancer Biology, Pharmacology, and Neuroscience, Meharry Medical College, Nashville, Tennessee, USA; 3 School of Graduate Studies, Meharry Medical College, Nashville, Tennessee, USA; 4 Department of Microbiology, Immunology, and Physiology, Meharry Medical College, Nashville, Tennessee, USA; 5 Department of Cancer Immunology and Virology, Dana-Farber Cancer Institute 1855, Boston, Massachusetts, USA; 6 Department of Medicine, Harvard Medical School 205260 , Boston, Massachusetts, USA; 7 Division of Infectious Diseases, Northwestern University Feinberg School of Medicine 12244, Chicago, Illinois, USA; 8 Department of Microbiology and Immunology, Loyola University Chicago 548051, Maywood, Illinois, USA; 9 Department of Pathology, Microbiology, and Immunology, Vanderbilt University Medical Center, Nashville, Tennessee, USA; St. Jude Children's Research Hospital, Memphis, Tennessee, USA

**Keywords:** cleavage and polyadenylation specificity factor 6 (CPSF6), human immunodeficiency virus (HIV), capsid, integration, preintegration complex

## Abstract

Cleavage and polyadenylation specificity factor 6 (CPSF6) is part of the cellular cleavage factor I mammalian (CFIm) complex that regulates mRNA processing and polyadenylation. CPSF6 also functions as an HIV-1 capsid (CA) binding host factor to promote viral DNA integration targeting into gene-dense regions of the host genome. However, the effects of CPSF6 on the activity of the HIV-1 preintegration complex (PIC)—the sub-viral machinery that carries out viral DNA integration—are unknown. To study CPSF6’s role in HIV-1 PIC function, we extracted PICs from cells that are either depleted of CPSF6 or express a mutant form that cannot bind to CA. These PICs exhibited significantly lower viral DNA integration activity when compared to the control PICs. The addition of purified recombinant CPSF6 restored the integration activity of PICs extracted from the CPSF6-mutant cells, suggesting a direct role of CPSF6 in PIC function. To solidify CPSF6’s role in PIC function, we inoculated CPSF6-depleted and CPSF6-mutant cells with HIV-1 particles and measured viral DNA integration into the host genome. A significant reduction in integration in these cells was detected, and this reduction was not a consequence of lower reverse transcription or nuclear entry. Additionally, mutant viruses deficient in CA–CPSF6 binding showed no integration defect in CPSF6-mutant cells. Finally, sequencing analysis revealed that HIV-1 integration into CPSF6-mutant cell genomes was significantly redirected away from gene-dense regions of chromatin compared to the control cells. Collectively, these results suggest that the CPSF6–CA interaction promotes PIC function both *in vitro* and in infected cells.

**IMPORTANCE:**

HIV-1 infection is dependent on the interaction of the virus with cellular host factors. However, the molecular details of HIV–host factor interactions are not fully understood. For instance, the HIV-1 capsid provides binding interfaces for several host factors. CPSF6 is one such capsid-binding host factor, whose cellular function is to regulate mRNA processing and polyadenylation. Initial work identified a truncated cytosolic form of CPSF6 to restrict HIV infection by blocking viral nuclear entry. However, it is now established that the full-length CPSF6 primarily promotes HIV-1 integration targeting into gene-dense regions of the host genome. Here, we provide evidence that CPSF6–CA interaction stimulates the activity of HIV-1 preintegration complexes (PICs). We also describe that disruption of CPSF6–CA binding in target cells significantly reduces viral DNA integration and redirects integration targeting away from gene-dense regions into regions of low transcriptional activity. These findings identify a critical role for the CPSF6–CA interaction in PIC function and integration targeting.

## INTRODUCTION

Approximately 82 million people have been infected with the human immunodeficiency virus 1 (HIV-1) (1). HIV-1 infects immune cells such as T lymphocytes, monocytes, and macrophages, which express CD4 receptor and CCR5 or CXCR4 co-receptors (2–8). Chronic HIV-1 infection results in the steady decline of CD4+ T cells and the development of acquired immunodeficiency syndrome (AIDS), a deadly disease that has already killed more than 42 million people worldwide (9, 10). Fortunately, highly effective antiretroviral therapy has transformed HIV-1 infection to a chronic disease and has dramatically reduced AIDS-related deaths. However, these therapies require lifelong adherence, have toxic side effects, and face drug resistance from the virus (4). Therefore, identification of novel therapeutic targets is a continuous need to effectively control HIV-1. Understanding the mechanisms by which host factors promote HIV-1 infection has the potential to identify such antiviral targets.

To begin infection, HIV-1 envelope glycoproteins bind to target cell receptor (CD4+) and co-receptor, causing fusion of the viral membrane with the cellular plasma membrane and the release of the viral capsid into the cytoplasm (11). The HIV-1 capsid contains two copies of a linear single-stranded (ss) viral RNA genome as well as other essential viral and host factors (12–17). The capsid shell is composed of two distinct capsomeres of the viral capsid (CA/p24) protein (18, 19): a major capsomere as a hexamer of CA, and a minor capsomere formed of a pentamer of CA (13, 20–22). The capsid shell is made up of ~200 CA hexamers and exactly 12 pentamers (14, 18, 23). The fullerene curvature of the HIV-1 capsid is formed by seven pentamers at the broad end and five pentamers at the narrow end. Although the exact mechanism of CA oligomerization is not fully understood, CA monomers are connected by specific CA–CA interactions that determine assembly and stability of the capsid. Contacts between the N-terminal domain (NTD)-NTD of CA monomers stabilize the hexamers and pentamers and form a central pore. The C-terminal domain of CA participates in dimeric and trimeric interactions between individual hexamers and pentamers. These CA interactions generate at least four known interfaces for host factor binding: (i) six hydrophobic clefts within each hexamer known as phenylalanine-glycine (FG) pockets, (ii) the arginine (R18) pore at the center of the hexamer, (iii) a cyclophilin A (CypA) binding loop on each CA-NTD subunit, and (iv) an electronegative patch formed at the vertex of three adjoining hexamers (18, 19, 24–35). Binding of host factors to these pockets of the capsid critically regulates HIV-1 infection (35).

Once in the target cell cytoplasm, the HIV-1 capsid is trafficked toward the nuclear pore complex (NPC) for nuclear entry (36, 37). Nuclear import of HIV-1 is dependent on the capsid (38–40) and is regulated by capsid-binding nucleoporins (Nups) of the NPC (41). The human NPC is constructed from ~33 Nups arranged in an eightfold rotational symmetry (42), and the HIV-1 capsid contains binding sites for several of these Nups. The large outer nucleoporin, Nup358 (a.k.a. RAN binding protein 2 or RanBP2), binds to the capsid via its cyclophilin-homology domain (19, 43, 44), which likely docks the capsid at the NPC (45–48). Early models indicated that the HIV-1 capsid would disassemble at the NPC to allow for translocation of the viral preintegration complexes (PICs) through the central channel (49). However, more recent work has suggested NPC plasticity can accommodate the capsid at its widest point (~60 nm) (50). Additionally, microscopy-based studies have suggested that HIV-1 capsids can remain intact during nuclear import (51, 52). Furthermore, NUP153, located at the nucleoplasmic side of the NPC, as well as the nuclear host factor cleavage and polyadenylation specificity factor 6 (CPSF6) display high affinity binding to the capsid lattice (48, 53), suggesting that at least some portion of the capsid is sustained during and after nuclear entry (51, 52, 54–57). Recent work has also helped to clarify the contributions of Nups to HIV-1 nuclear transport. About one-third of human Nups are enriched for FG dipeptide repeats and are accordingly referred to as FG-Nups. Several FG-Nups, including Nup62, Nup98, Nup58, Nup42, POM121, and Nup153, bind the HIV-1 capsid FG pocket (41, 58–60). CPSF6 also harbors a single FG dipeptide that binds this same region of the capsid (61, 62).

While en route to the nucleus, the capsid core-associated reverse transcription complex (RTC), containing the viral reverse transcriptase (RT) and integrase (IN) enzymes, synthesizes a double-stranded DNA copy of the viral ssRNA genome (37, 63). IN-mediated processing of the viral DNA ends operationally morphs the RTC into PIC (64). PICs were first described as high-molecular-weight sub-viral nucleoprotein complexes in cell extracts that integrated reverse-transcribed viral DNA into an exogenous target DNA *in vitro* (65, 66). Aided by host factors, the PIC integrates viral DNA preferentially into actively transcribed genes to establish a provirus that serves as the genetic element to produce progeny virions (64). CPSF6 has emerged as a critical host factor that promotes post-import trafficking of HIV-1 PICs and integration targeting of the viral DNA into gene-dense regions of host chromosomes (35).

CPSF6 was first implicated in HIV-1 biology via a genetic screen for mouse-specific restriction factors (67), as mouse cells are refractory to HIV-1 infection (68). This study identified a C-terminal truncated CPSF6, called CPSF6-358, which binds the HIV-1 capsid (67). CPSF6 is conserved among mammals and is involved in mRNA processing and polyadenylation as part of the cellular pre-mRNA processing complex cleavage factor I mammalian (CFIm) (69). In humans, *CPSF6* is located on chromosome 12 (12q15) and encodes a predominant 551-amino acid (aa) splice variant, which is associated with nuclear paraspeckles. Structurally, CPSF6 has three key regions. Near the N-terminus (aa 81–157) is the RNA recognition motif (RRM) that binds to CPSF5 in the CFIm complex (70, 71). The C-terminal (aa 489–551) arginine/serine-like domain (RSLD) engages the β-karyopherin transportin 3 (a.k.a. TRN-SR2) and acts as the nuclear localization signal for CPSF6 (72, 73). The RSLD also displays liquid-liquid phase separation activity *in vitro* (74) and confers higher-order capsid binding, which is critical for HIV-1 capsid to move downstream from the nuclear rim during infection (75). The central region of CPSF6 (aa 208–398) harbors a proline-rich domain with a prion-like low complexity region (aa 217–326), including the FG dipeptide that directly engages with the HIV-1 capsid (53, 62).

The role of CPSF6 in HIV-1 replication is multipronged. Initially, CPSF6-358 lacking the RSLD was shown to inhibit HIV-1 nuclear import (67). Passaging experiments in the presence of CPSF6-358 produced an HIV-1 mutant bearing an N74D substitution in CA, which was resistant to CPSF6-358-mediated inhibition (67, 76). These results indicated that CA was the viral determinant for CPSF6-358 restriction (67, 76). A separate mutation near the N74 residue, A77V, was also reported to reduce CPSF6 interaction with the HIV-1 CA (77). CPSF6 depletion can marginally inhibit HIV-1 nuclear import (78) and, in monocyte/macrophages, CPSF6 protects HIV-1 replication complexes from innate immune sensing (79, 80). However, CPSF6 appears to primarily regulate intranuclear localization of HIV-1 PICs for integration site selection (78, 81, 82). For instance, CPSF6 enables trafficking of HIV-1 cores to colocalize with nuclear speckles for PIC-mediated integration into speckle-associated domains (SPADs) of the human genome (75, 83). CPSF6 also seems to coordinate with other capsid-binding host factors, such as CypA, to orchestrate the early steps of HIV-1 infection (84). However, the mechanism by which CPSF6 regulates PIC function has not been previously studied.

Here, we used human cell lines depleted of CPSF6 or expressing a CPSF6 mutant (FG > AA) that cannot bind to HIV-1 CA (53) for PIC-based experiments. Such PICs exhibited significantly lower integration activity compared to PICs isolated from matched control cells, whereas the addition of purified CPSF6 protein stimulated PIC activity. To study the effects of CPSF6 on PIC function during HIV-1 infection, we quantified DNA replication intermediates (reverse transcription, 2-long terminal repeat [2-LTR] circles for nuclear entry, and integration) in HIV-1-infected cells and evaluated integration site selection profiles. Our results showed that disrupting CPSF6–CA binding reduced HIV-1 integration without measurably reducing reverse transcription or nuclear entry. Furthermore, the CPSF6–CA binding-deficient mutant viruses N74D and A77V showed minimal integration defects in the CPSF6-mutant cells. We also found that disruption of CPSF6–CA binding significantly retargeted viral DNA integration into lamin-associated domains (LADs) instead of gene-dense SPADs. These results, for the first time, support a direct role of CPSF6 in PIC function.

## MATERIALS AND METHODS

### Chemicals, plasmids, and other reagents

The antiretroviral compound raltegravir (RAL) was obtained from BEI Resources (NIH HIV Reagents Program; Manassas, VA) and prepared as previously described (85). The virus particles utilized in this study were generated from the HIV-1 molecular clone pNLX.Luc(R-)ΔAvrII (86), and its CA mutant derivatives CA-N74D and CA-A77V (86). The plasmid pHCMV-G encoding vesicular stomatitis virus G protein VSV-G (87) was used in co-transfection experiments for generating pseudotyped HIV-1 particles. Recombinant CPSF6 protein expressed in *Escherichia coli* strain BL21(DE3) was purified as previously described (75).

### Western blot

To detect proteins by Western blot, cellular lysates were prepared using 1× RIPA buffer (Sigma-Aldrich) and total protein concentrations were quantified by bicinchoninic acid (BCA) protein assay (Pierce). Equal amounts of total protein from the lysates were electrophoresed on 4%–12% sodium dodecyl sulfate (SDS)-polyacrylamide gels and transferred to nitrocellulose membranes using a semidry blotter (Bio-Rad). Membranes were blocked with 5% (wt/vol) nonfat milk in Tris-buffered saline with Tween 20 (TBST; pH 8.0; Chem Cruz). Blots were then probed with the primary antibody in blocking buffer (anti-CPSF6: Proteintech catalog # 15489-1-AP at 1:4,000 dilution or Abcam ab175237 at 1:10,000; anti-actin: Sigma-Aldrich catalog # A2228 at 1:60,000 dilution or A3854-200UL at 1:20,000) and subsequently probed with a secondary antibody conjugated to horseradish peroxidase (anti-rabbit: Bio-Rad catalog # 1706515 at 1:6,000 dilution or Dako P0448 at 1:10,000; Bio-Rad catalog # 1706516 anti-mouse at 1:40,000 dilution). All blots were washed in TBST and developed by using the enhanced chemiluminescence procedure (Bio-Rad). Densitometry analysis was performed by using LI-COR Image Studio Digits version 5.2 software (LI-COR). Data were normalized to levels of β-actin.

### Cell culture

TZM-bl, human embryonic kidney 293T (HEK293T), HeLa, Jurkat, and SupT1 cell lines were obtained from the American Type Cell Culture Collection (Manassas, VA). The TZM-bl and HEK293T cells were cultured in Dulbecco’s Modified Eagle’s medium (Thermo Fisher Scientific) containing 10% heat-inactivated fetal bovine serum (FBS), penicillin (50 IU/mL), and streptomycin (50 µg/mL). Jurkat and SupT1 cells were cultured in Roswell Park Memorial Institute 1640 medium (Thermo Fisher Scientific) containing 10% heat-inactivated FBS, penicillin (50 IU/mL), and streptomycin (50 µg/mL). All cell lines were cultured at 37°C with 5% CO_2_.

### CRISPR-Cas9 knockout and knock-in

Generation of CPSF6 knockout (CKO) T cell lines was attempted using a CRISPR-Cas9-based knockout strategy (88, 89). HEK293T and HeLa CKO cell lines were previously described (53, 82, 90, 91). CPSF6 knock-in (CKI) SupT1 cells were generated by mutating the portion of CPSF6 that encodes for the capsid-binding FG motif (FG > AA). For this, a single-guide RNA (sgRNA) 5′-CCCAATGGAGGCTGCCCAAA-3′ targeting exon 7 of *CPSF6* and a single-stranded oligodeoxynucleotide (ssODN) donor template 5′-GCCCTCCACCACCAGTTCTTTTTCCTGGACAACCTGCTGCGCAGCCTCCATTGGGTCCACTTCCTCCTGGCCCTC-3′ were purchased from Horizon Discovery. To form CRISPR ribonucleoproteins (crRNPs), 5 µL of 60 µM CPSF6 sgRNA and 5 µL of 30 µM TrueCut Cas9 v2 (Thermo Fisher) were mixed and incubated at 37°C for 15 min. Approximately 1 × 10^6^ SupT1 cells were transfected with 10 µL of 15 µM crRNPs and 10 µL of 100 µM ssODN using the nucleofection kit and Nucleofector I device (Lonza), following the manufacturer’s protocol. Three to 5 days post-transfection, single cells were isolated by limiting dilution cloning in 96-well plates and then transferred to 24-well plates as the cells propagated. To confirm biallelic knock-in, genomic DNA was extracted, and the targeted locus was amplified using primers 5′-TGGCACGAATTCGTAAGGATATACTTCATTGTAGTTGGTAGTG-3′ and 5′-CACTGAGGATCCGCGTTCTTGCAGTATCCATTTCC-3′. Sanger sequencing of PCR amplicons using primer 5′-CCATAGTCACCCCTATCATATGG-3′ was performed before and after cell cloning.

### Viral stock preparation

The viruses utilized in this study were generated from HIV-1 molecular clone pNLX.Luc(R-)ΔAvrII (wild type [WT]) and CA mutant derivatives (N74D and A77V). Infectivity and viral DNA quantification experiments were performed with VSV-G-pseudotyped NL4-3 virions (referred to as HIV-1.Luc) that were generated from the pNLX.Luc(R-)ΔAvrII plasmid that contains a frameshift in the *env* gene and the firefly luciferase-coding sequence inserted in place of the *nef* gene (86). All virus stocks were prepared as previously described (85). Briefly, 5 × 10^6^ HEK293T cells were seeded in 10 cm cell culture plates and cultured overnight. The next day, cells in each culture plate were co-transfected with 10 µg of pNLX.Luc(R-)ΔAvrII and pHCMV-G plasmid DNA complexed with 50 µL polyethylenimine (PEI) reagent (1 mg/mL) (Sigma-Aldrich catalog # 764604), and the culture media was changed 16 h post-transfection. Forty-eight hours later, the virus-containing culture media was collected, subjected to low-speed centrifugation, and the supernatant was passed through a 0.45 µm filter. The filtrate was treated with DNase I (Thermo Fisher Scientific catalog #AM2238) at a final concentration of 20 µg/mL at 37°C. The viral stocks were stored at −80°C until assayed for infectivity. Subsequently, an enzyme-linked immunosorbent assay (ELISA) was used to assay the virus preparations for p24 amounts using anti-p24 antibody as previously described (85). Virus infectivity was determined using TZM-bl indicator cells, as described previously (92).

### Single-cycle infection assay

A total of 1 × 10^6^ SupT1 cells per well in a 12-well flat-bottom cell culture plate was spinoculated (480 × *g*) with 450 ng of pseudotyped HIV-1.Luc for 2 h at 25°C and was then cultured for 24 h in a 37°C/5% CO_2_ incubator. Subsequently, cells were pelleted (500 × *g*) and washed three times with phosphate-buffered saline (PBS, pH 7.4, Thermo Fisher Scientific). Cell samples resuspended in PBS were aliquoted for assessing infectivity via luciferase assay and for total DNA isolation. For luciferase assays, cell pellets were lysed in 100 µL 1× Glo Lysis Buffer (Promega catalog # E2661), rocked for 15–30 min at 25°C, and centrifuged (16,000 × *g*) for 5 min to pellet cellular debris. Triplicate 25 µL aliquots of each sample supernatant were dispensed into 96-well black flat-bottom plates. Luciferase activity was determined using the Luciferase assay system (Promega) in a microplate reader (Biotek). Infectivity assays for HEK293T cells were performed by seeding 1 × 10^6^ cells in each well of a 12-well flat-bottom cell culture plate 24 h prior to the addition of pseudotyped HIV-1.Luc. Cells were inoculated with viral particles and spinoculated for 2 h at 25°C prior to incubation in a 37°C/5% CO_2_ incubator for 24 h. Cells were then harvested by detachment from the plate and pelleted by centrifugation (500 × *g*). Cell samples were processed for luciferase assay as described above for SupT1 cells.

### Total DNA isolation for qPCR assays

To isolate total DNA from infected cells, 1 × 10^6^ SupT1 cells in each well of a 12-well flat-bottom cell culture plate were spinoculated (480 × *g*) with 450 ng of pseudotyped HIV-1.Luc for 2 h at 25°C and were then cultured for 24 h in a 37°C/5% CO_2_ incubator. Half of the cells were used for assessing virus infectivity via a luciferase reporter assay, and total DNA was isolated from the remaining cells by using the Quick-DNA miniprep kit (Zymo Research).

### qPCR for measuring reverse transcription and 2-LTR circles

Late-stage reverse transcription products were quantified by using SYBR Green-based qPCR, and 2-LTR circles were quantified by using TaqMan probe-based qPCR. The SYBR Green-based qPCR mix contained 1× iTaq Universal SYBR Green Supermix (Bio-Rad), 300 nM concentrations (each) of forward primer (5′-TGTGTGCCCGTCTGTTGTGT-3′) and reverse primer (5′-GAGTCCTGCGTCGAGAGAGC-3′), and 100 ng of total DNA from infected cells. The TaqMan probe-based qPCR mix contained 1× iTaq Universal Probe Supermix (Bio-Rad), 300 nM (each) of forward primer (5′-AACTAGGGAACCCACTGCTTAAG-3′) and reverse primer (5′-TCCACAGATCAAGGATATCTTGTC-3′), 100 nM of the TaqMan probe (5´-[FAM] ACACTACTTGAAGCACTCAAGGCAAGCTTT-[TAMRA]−3´), and 100 ng of total DNA from infected cells. The cycling conditions for SYBR Green-based qPCR included an initial incubation at 95°C for 3 min, followed by 39 cycles of amplification and acquisition at 94°C for 15 s, 58°C for 30 s, and 72°C for 30 s. For the SYBR Green-based qPCR, the thermal profile for melting curve analysis was obtained by holding the sample at 65°C for 31 s, followed by a linear ramp in temperature from 65 to 95°C with a ramp rate of 0.5°C/s and acquisition at 0.5°C intervals. The TaqMan probe-based qPCR included an initial incubation at 95°C for 3 min, followed by 39 cycles of amplification and acquisition at 94°C for 15 s, 58°C for 30 s, and 72°C for 30 s. During qPCR of the samples, a standard curve was generated in parallel and under the same conditions using 10-fold serial dilutions of known copy numbers (10^0^ to 10^8^) of the pNLX.Luc(R-)ΔAvrII plasmid (for late reverse transcription products) or the p2LTR plasmid containing the 2-LTR junction sequence (for 2-LTR circles). CFX Manager software (Bio-Rad) was used to analyze the data and determine the copy numbers of the late reverse transcription products and the 2-LTR circles by plotting the data against the respective standard curves.

### Alu-gag nested qPCR for measuring HIV-1 proviral integration

To measure integrated HIV-1 proviral DNA, a nested PCR method, involving a first-round endpoint PCR with primers designed to amplify the junctions of chromosomally integrated viral DNA with host DNA, followed by a second round of qPCR with primers that amplify viral LTR-specific sequences present in the first-round PCR amplicons, was used with some modifications. Briefly, the first-round PCR was performed in a final volume of 50 µL containing 100 ng of total DNA from infected cells, 1× Phusion reaction buffer (NEB), deoxy-nucleotide triphosphate (dNTP) nucleotide mix containing 200 µM concentrations of each nucleotide (Promega), 500 nM (each) of primers that target the host chromosomal Alu repeat sequence (5′-GCCTCCCAAAGTGCTGGGATTACAG-3′) and HIV-1 Gag sequence (5′-GTTCCTGCTATGTCACTTCC-3′), and 1.25 U of Phusion DNA Polymerase (NEB). The thermocycling conditions included an initial incubation at 95°C for 5 min, followed by 23 cycles of amplification at 94°C for 30 s, 50°C for 30 s, 72°C for 4 min, and a final incubation at 72°C for 10 min. The second-round qPCR mix contained a 1/10 volume of the first-round PCR products as the template DNA, 1× iTaq Universal Probe Supermix (Bio-Rad), 300 nM (each) of the viral LTR-specific primers that target the R region (5′-TCTGGCTAACTAGGGAACCCA-3′) and the U5 region (5′-CTGACTAAAAGGGTCTGAGG-3′), and a 100 nM concentration of TaqMan probe (5´-[6-FAM]-TTAAGCCTCAATAAAGCTTGCCTTGAGTGC-[TAMRA]−3´). The qPCR cycling conditions included an initial incubation at 95°C for 3 min, followed by 39 cycles of amplification and acquisition at 94°C for 15 s, 58°C for 30 s, and 72°C for 30 s. During the second round of qPCR of the samples, a standard curve was generated in parallel and under the same conditions using 10-fold serial dilutions of known copy numbers (10^0^ to 10^8^) of the pNLX.Luc(R-)ΔAvrII plasmid. Data were analyzed using CFX Manager software (Bio-Rad), and integrated viral DNA copy numbers were determined by plotting the qPCR data against the standard curve.

### Extraction of HIV-1 PICs

HIV-1 PICs were extracted from infected T cells, HEK293T, and HeLa cells using a modified version of previously published methods (85, 93–95). A total of 8 × 10^6^ cells was distributed equally in six-well plates in HIV-1-containing media (30 mL of 450 ng/mL of HIV-1 p24) and subsequently spinoculated at 480 × *g* for 2 h at 25°C. Cells were then cultured for 5 h in a 37°C/5% CO_2_ incubator. After culturing, cells were pelleted by centrifugation for 10 min (300 × *g*). The supernatant was then carefully aspirated, and the cell pellet was washed twice with 2 mL of K−/− buffer (20 mM HEPES [pH 7.6], 150 mM KCl, 5 mM MgCl_2_) at room temperature. The pellet was then gently lysed by resuspending in 2 mL of ice-cold K+/+ buffer (20 mM HEPES [pH 7.6], 150 mM KCl, 5 mM MgCl_2_, 1 mM dithiothreitol, 20 µg/mL aprotinin, 0.025% [wt/vol] digitonin) and rocking for 10 min at room temperature. The cytoplasmic extract containing the PICs was then separated from other cellular components by differential centrifugation for 4 min at 1,500 × *g* at 4°C. The supernatant was transferred to a new microcentrifuge tube and centrifuged for 1 min at 16,000 × *g* at 4°C. The resulting supernatant was transferred to a new microcentrifuge tube and treated with RNase A (Thermo Fisher Scientific) for 30 min at room temperature to remove any cellular and or viral RNA. Finally, 60% sucrose (wt/vol) in K−/− buffer was added to a final concentration of 7% and gently mixed by pipetting. These PICs were then aliquoted, flash frozen in liquid nitrogen, and then stored in a −80°C freezer.

### PIC-associated integration activity measurements

*In vitro* integration assays were performed using a modified version of the published protocol (96). The target DNA used was a PCR-amplified 2 kb region of the phi-X174 genome. To construct this fragment, PCR was performed in a final volume of 50 µL containing 50 ng of the phi-X174 plasmid (Promega), 500 nM concentrations (each) of the forward primer (5´-CGCTTCCATGACGCAGAAGTT- 3´) and the reverse primer (5´-CACTGACCCTCAGCAATCTTA-3´), 1× Phusion reaction buffer (NEB), dNTP nucleotide mix containing 200 µM concentrations of each nucleotide (NEB), and 1.25 U of Phusion DNA Polymerase (NEB) under the following thermocycling conditions: initial incubation at 95°C for 2 min, followed by 34 cycles at 95°C for 30 s, 53°C for 30 s, and 72°C for 2 min, and a final incubation at 72°C for 10 min. PCR products were resolved by standard agarose gel electrophoresis, and the phi-X174-specific PCR amplicon was gel purified by using a Gel DNA Recovery Kit (Zymo Research).

The *in vitro* integration reaction was carried out by mixing 200 µL of PICs and 600 ng of target DNA and then incubating the mixture at 37°C for 45 min. The integration reaction was stopped and deproteinized by adding SDS, EDTA, and proteinase K to final concentrations of 0.5%, 8 mM, and 0.5 mg/mL, respectively, followed by incubation overnight at 56°C. The deproteinized sample was mixed with an equal volume of phenol (equilibrated with 10 mM Tris-HCl), mixed thoroughly by vortexing, and centrifuged (16,000 × *g*) for 2 min at room temperature. The aqueous phase was extracted once with an equal volume of phenol:chloroform (1:1) mixture, followed by an equal volume of chloroform. The DNA was precipitated by adding 2.5 volumes of 100% ice-cold ethanol in the presence of sodium acetate (0.3 M, final concentration) and the coprecipitant glycogen (25 to 100 µg, final concentration), followed by an incubation overnight at −80°C. The sample was centrifuged (16,000 × *g*) for 30 min at 4°C, and the resultant DNA pellet was washed once with 80% ethanol using centrifugation (16,000 × *g*) for 10 min at 4°C. The precipitated DNA was air dried at room temperature, resuspended in 50 µL of nuclease-free water, and used as the template DNA in a nested qPCR. A first-round standard PCR, designed to amplify only the integrated virus-target DNA junctions, was carried out in a final volume of 50 µL containing 5 µL of purified DNA product from the integration reaction, 500 nM concentrations (each) of primers targeting the target DNA (5′-CACTGACCCTCAGCAATCTTA-3′) and the viral LTR (5′-GTGCGCGCTTCAGCAAG-3′), 2× Megafi Reaction Buffer (ABM), and 1.25 U of MegaFi DNA polymerase (ABM) under the following thermocycling conditions: initial incubation at 95°C for 5 min, followed by 23 cycles at 94°C for 30 s, 55°C for 30 s, and 72°C for 2 min, and a final incubation at 72°C for 10 min. The second-round qPCR designed to amplify only the viral LTR-specific region contained a 1/10 volume of the first-round PCR products as the template DNA, 1× iTaq Universal Probe Supermix (Bio-Rad), 300 nM concentrations (each) of the viral LTR-specific primers that target the R region (5´-TCTGGCTAACTAGGGAACCCA-3´) and the U5 region (5′-CTGACTAAAAGGGTCTGAGG-3′), and a 100 nM concentration of TaqMan probe (5´-[6-FAM]-TTAAGCCTCAATAAAGCTTGCCTTGAGTGC-[TAMRA]-3´). The qPCR run included an initial incubation at 95°C for 3 min, followed by 39 cycles of amplification and acquisition at 94°C for 15 s, 58°C for 30 s, and 72°C for 30 s. During qPCR of the samples, a standard curve was generated in parallel and under the same conditions using 10-fold serial dilutions of known copy numbers (10^0^ to 10^8^) of the HIV-1 molecular clone plasmid. Data were analyzed using CFX Manager software (Bio-Rad), and integrated viral DNA copy numbers were determined by plotting the qPCR data against the standard curve. To determine the integration efficiency (i.e., ratio of chromosome-integrated viral DNA copy numbers to corresponding PIC-associated viral DNA copy numbers) of the *in vitro* integration reactions, the PIC-associated viral DNA was isolated and copy numbers were determined by qPCR.

### Integration site selection analysis

Genomic DNA (~5 µg) isolated from infected cells was digested overnight with a cocktail of enzymes (MseI and BglII; 100 U each). Following overnight ligation (four parallel reactions) to asymmetric linkers, DNA was purified using a PCR purification kit. The ligated samples were subjected to two rounds of ligation-mediated PCR using virus and linker-specific primers. Following PCR purification, libraries were assessed for fragment size distribution by TapeStation 4150, quantified by DNA fluorimetry (Qubit), and then pooled at 10 nM. Pooled samples were further diluted to 2 nM in Illumina sequencing resuspension buffer (RSB, Ref # 20762979). Next, PhiX Control v3 DNA was spiked at 30%, and the sample was diluted to 650 pM with RSB. The mixture (20 µL) was loaded into a P1 300 cycle cartridge and sequenced on an Illumina NextSeq 2000 sequencer. Raw fastq files were demultiplexed using the Sabre tool or by a Perl script (97). Post demultiplexing, files were trimmed, aligned to the human genome build hg19, and bed files were generated as described (75, 90). Integration into genes and SPADs was scored as within these genomic coordinates. For transcription start sites (TSSs), regions enriched with a high proportion of C/G nucleotides (CpG islands), and LADs, sites were mapped within ±2.5 kb windows (5 kb surrounding these coordinates). Gene density was assessed as the number of genes per Mb. Random integration controls (RICs) were generated by shearing hg19 *in silico* using the restriction enzyme sites used to generate the wet bench samples and then mapping the resultant fragments with respect to the aforementioned genomic annotations (90).

### Statistical analyses

Most infection and PIC experiments were conducted at least three times with triplicate samples. Data were expressed as mean ± standard error of the mean (SEM) obtained from three independent experiments. Significance of differences between control and treated samples were determined by paired, two-tailed Student’s *t*-test. Duplicate infections were conducted for integration site mapping experiments. Differences between test samples versus WT and RIC values were generally assessed by Fisher’s exact test. For gene density, the Wilcoxon rank-sum test was used to assess statistical differences. For all statistical assessments, *P*-values of <0.05 were considered statistically significant.

## RESULTS

### CPSF6 depletion reduces HIV-1 PIC-associated viral DNA integration activity

**Fig 1:**
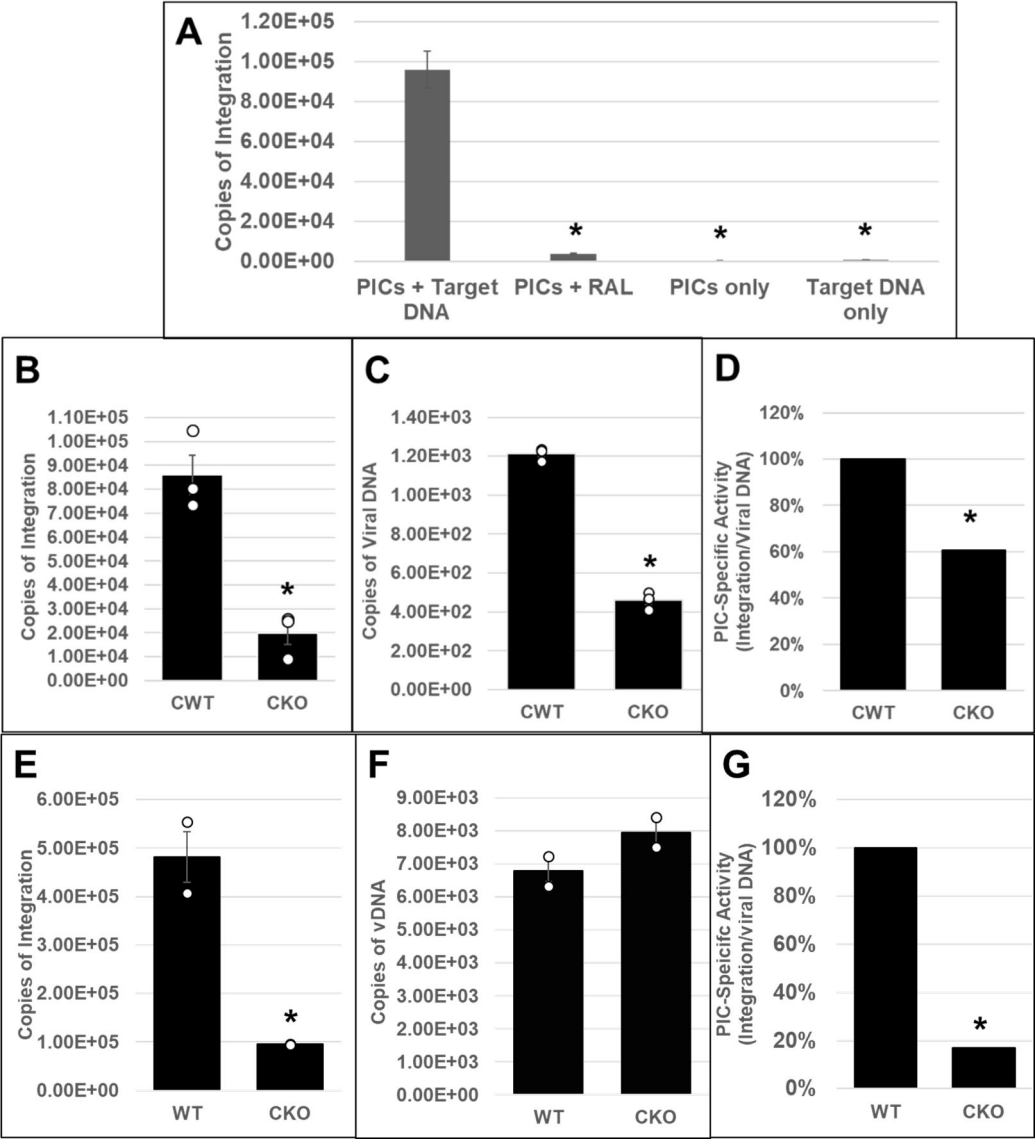
Effect of CPSF6 depletion on HIV-1 PIC integration activity *in vitro*. (**A–D**) HEK293T CPSF6 WT (CWT) and CKO cells were spinoculated with 450 ng p24 virus/million cells as described in Materials and Methods. PICs were extracted, and *in vitro* integration reactions were carried out using phi-X174 target DNA. Reactions in the presence of the IN inhibitor RAL or in the absence of the target DNA served as controls. A nested qPCR-based approach was utilized to measure the integration activity of the PICs. (**A**) Assessment and verification of PIC integration activity. (**B**) Comparison of *in vitro* integration activity of PICs extracted from CWT or CKO cells. (**C**) Copies of PIC-associated viral DNA determined via qPCR. (**D**) PIC-specific activity normalized to the amount of viral DNA in the different preps (integration/viral DNA). Specific activity is displayed as percentage relative to CWT cell PICs. (**E–G**) PIC activity from CKO and WT HeLa cells. (**E**) PIC-associated integration activity. (**F**) Viral DNA content of the PICs. (**G**) Specific PIC activity. White circles in graphs represent separate biological replicates. Error bars represent SEM. The *P*-values (*) represent statistical significance (*P* < 0.05) between control and CKO PICs.

CPSF6 binds to the HIV-1 capsid and plays multiple roles during the early steps of infection (35, 67). However, the dominant function of CPSF6 seems to enable post-nuclear import localization of HIV-1 PICs to actively transcribing genes for viral DNA integration (74, 79, 81–83). However, the mechanism by which CPSF6 regulates integration site selection is not fully understood. Particularly, the effects of CPSF6 on PIC function have not been previously reported. PICs are sub-viral nucleoprotein complexes consisting of the viral IN, viral DNA, and other viral/host factors that carry out viral DNA integration to establish infection (64). Importantly, PICs can be extracted from infected cells, and these PICs retain viral DNA integration activity *in vitro* (98, 99). Therefore, to study CPSF6’s effect on PIC function, we first inoculated HEK293T (82) and HeLa CKO cells (91), as well as matched control cells, with cell-free DNase I-treated VSV-G-pseudotyped HIV-1 particles. Then, cytoplasmic extracts containing the PICs were prepared (85, 93, 96) and subjected to an *in vitro* integration assay containing a linear double-stranded target DNA substrate. Then, the copies of viral DNA integrated into the target DNA were quantified by a nested qPCR method (85, 93, 100). As expected, the PICs from the infected control cells efficiently carried out viral DNA integration, and the integration activity was inhibited by the IN inhibitor RAL (Fig. 1A). Moreover, measurable integration activity was not detected in reactions lacking PICs or target DNA, confirming the specificity of the assay. Our results further revealed that PICs isolated from CKO HEK293T cells exhibited significantly lower integration activity (a fourfold decrease) when compared to the PICs from the control cells (Fig. 1B). Quantification of the viral DNA levels in these PIC preparations revealed lower viral DNA in the PICs from CKO cells compared to the control cells (Fig. 1C). Therefore, we determined PIC-specific activity by calculating the ratio of the copies of integrated viral DNA to the copies of PIC-associated viral DNA (Fig. 1D). These calculations indicated that the specific integration activity of PICs from CKO HEK293T cells was significantly lower (~40%) compared to those from the control cells. Similar defects in PIC activity were exhibited using extracts of CKO HeLa cells (Fig. 1E through G). For example, PICs from CKO HeLa cells showed a significantly lower integration activity (almost ~4- to 5-fold decrease) when compared to the PICs from the control cells (Fig. 1E). The viral DNA levels in the PICs of the WT and CKO HeLa cells were minimally changed (Fig. 1F), in contrast to the lower viral DNA levels in the HEK293T CKO cells (Fig. 1C). While a recent study reported comparable viral DNA levels in HeLa-CKO cells and the matched control cells (91), HEK293T-CKO cells have been reported to support marginally higher viral DNA levels than the matched control cells (82). Notably, these previous studies used total cellular DNA isolated from infected cell samples as template DNA for measuring viral DNA levels by qPCR. In contrast, our measurements detected PIC-associated viral DNA levels (Fig. 1) in cytoplasmic extracts prepared at 5 h post-infection (hpi). Whether this technical difference in the qPCR template DNA used and/or the timing of viral DNA measurements contributed to the lower levels observed in HEK293T-CKO cells at 5 hpi requires further investigation. Nevertheless, PIC-specific activity calculations revealed significant reductions in integration activities, regardless of whether PICs were isolated from CKO HEK293T (Fig. 1C) or CKO HeLa cells (Fig. 1G). Collectively, these biochemical studies demonstrate that PICs extracted from CPSF6-depleted cells exhibit lower viral DNA integration activity *in vitro* and suggest that CPSF6 is critical for HIV-1 PIC function.

### Disruption of CPSF6–CA interaction reduces HIV-1 PIC-associated viral DNA integration activity

Our studies of PICs from CPSF6-depleted HEK293T and HeLa cells supported a functional role of CPSF6 in PIC activity. However, these cell types are not permissive to HIV-1 infection. Therefore, we next sought to study PICs using a T cell line model, which is arguably more physiologically relevant for HIV-1 studies. Previous studies have described technical challenges associated with generating CKO T cell lines (90, 101). Accordingly, renewed efforts here to generate CKO T cell lines using CRISPR-Cas9 approaches were also unsuccessful (data not shown). As a workaround, we adopted a CRISPR-Cas9-based microhomology-mediated end-joining strategy (102) to mutate the region of *CPSF6* exon 7 encoding the CA-binding FG motif to alanine-alanine (AA) (Fig. S1A and B). We chose SupT1 cells to generate CKI cell lines due to the long-standing usage of this T cell line in HIV-1 PIC studies (65, 66, 85, 93–95, 98, 99, 103, 104). Following nucleofection of crRNPs and a single-stranded DNA repair template, cells were cloned by limiting dilution, and individual cell clones were expanded and analyzed for the site-directed mutations. A second set of cell clones was derived under identical conditions using non-targeting-guide RNA as controls. Characterization of the SupT1 WT and CKI cell lines showed minimal differences in CPSF6 expression (Fig. S1C) and cell viability (Fig. S2).

**Fig 2:**
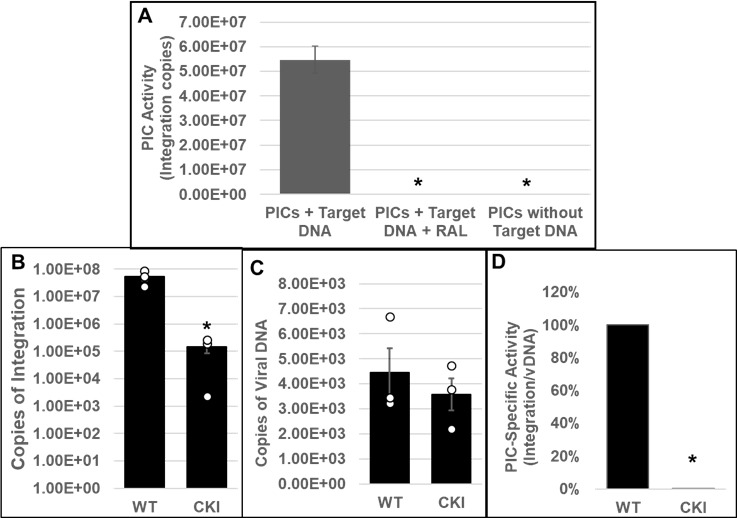
Effect of disrupting the CPSF6–CA interaction on the *in vitro* integration activity of HIV-1 PICs. (**A**) Parental WT SupT1 cells or CKI cells were spinoculated with high titer infectious HIV-1 particles (1,500 ng p24/mL) and cultured for 5 h followed by extraction of PIC-containing cytoplasmic extracts. *In vitro* assays using these PICs as the source of integration activity and quantification of viral DNA integration into an exogenous target DNA by nested PCR were carried out with appropriate controls. (**B**) *In vitro* integration assays of PICs extracted from WT1 and from CKI cells were carried out, and the copy numbers of integrated viral DNAs were determined. (**C**) Viral DNA copy numbers were quantified by qPCR. (**D**) The specific activity of the PIC-mediated integration was determined by calculating the ratio of integrated viral DNA copy numbers (from panel B) to the corresponding PIC-associated viral DNA copy numbers (from panel C). Mean values from two independent experiments, each conducted in triplicate, are shown with error bars representing the SEM. White circles present in graphs represent individual data points. The *P*-values (*) represent statistical significance (*P* < 0.05) between the control and CKI PICs.

PICs extracted from WT and CKI clonal cell lines inoculated with HIV-1 particles were subjected to our *in vitro* integration assay. Measurement of viral DNA integration revealed that the PICs from the WT SupT1 cells efficiently integrated the viral DNA into the exogenous target DNA, and this activity was significantly reduced in the presence of RAL (Fig. 2A). Notably, the PICs from the WT SupT1 cells exhibited significantly higher integration activity, almost 2-log-fold higher, when compared to the PICs from CPSF6 WT (CWT) HEK293T cells (Fig. 1A) and CWT HeLa cells (Fig. 1E). However, the PICs from the CKI SupT1 cells exhibited significantly lower integration activity (~2-log defect) when compared to the PICs from the WT SupT1 cells (Fig. 2B). This level of reduction was in stark contrast to the ~5-fold reductions in integration activity observed with the PICs from CKO HEK293T and CKO HeLa cells (Fig. 1B and E). To assess whether the lower integration activity of PICs from CKI SupT1 cells was due to a reduction in the number of PICs formed in the CKI cells, we quantified the amount of viral DNA in the PIC preparations. We found that the viral DNA levels in the CKI cells were not significantly different from that of the control cells (Fig. 2C). Accordingly, estimation of PIC specific activity by calculating the ratio of the copies of integrated viral DNA to the copies of PIC-associated viral DNA indicated that the integration activity of PICs of CKI cells was significantly lower (>95%) compared to the control cell complexes (Fig. 2D). Together, our results in Fig. 1 and 2 provide *in vitro* biochemical evidence that PICs extracted from cells depleted of CPSF6 or cells that express a mutant CPSF6 that cannot bind CA exhibit significantly lower integration activity.

### Addition of CPSF6 enhances the integration activity of HIV-1 PICs

**Fig 3:**
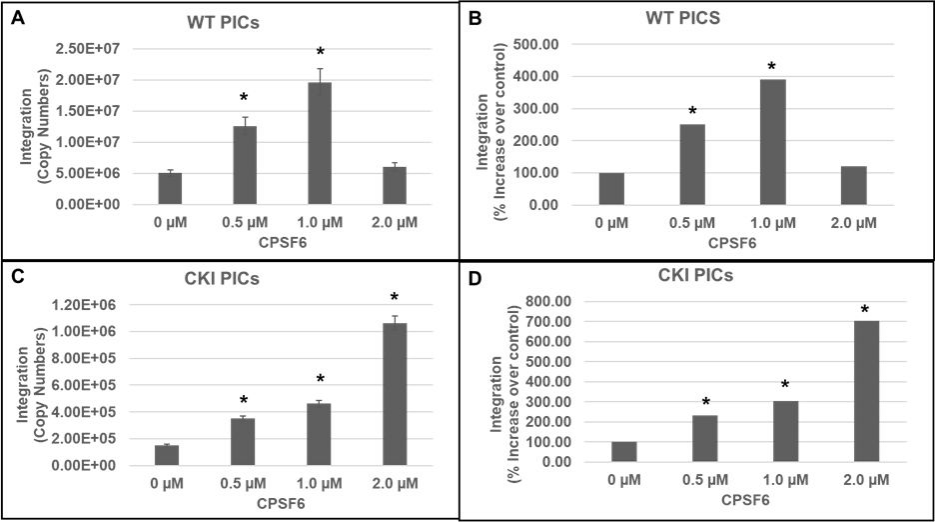
Effects of CPSF6 protein on PIC-associated integration activity. *In vitro* integration activity of PICs extracted from WT control cells (**A and B**) and from CKI cells (**C and D**) was assessed in the absence or presence of varying concentrations (0 µM–2 µM) of purified recombinant CPSF6 protein. (**A and C**) The copy number of the integrated viral DNAs was determined by nested qPCR. (**B and D**) Integration activity plotted as fold change relative to the integration activity of the respective control PICs. Mean values from two independent experiments, each conducted with triplicate samples, are shown with error bars representing the SEM. The *P*-values (*) represent statistical significance (*P* < 0.05) relative to untreated PICs (0 µM).

Our integration activity measurements suggested that CPSF6 is important for PIC function (Fig. 1 and 2). To pinpoint whether CPSF6 directly regulates PIC-mediated viral DNA integration, we examined the effect of the addition of purified recombinant CPSF6 protein to *in vitro* integration reactions (Fig. 3). CPSF6 protein was added at increasing concentrations (0, 0.5, 1, and 2 µM) to the reaction mixtures containing PICs extracted from WT or CKI SupT1 cells. Quantification of viral DNA integration showed that the addition of CPSF6 significantly stimulated the activity of PICs from both the WT (Fig. 3A and B) and CKI cells (Fig. 3C and D). Because of the larger number of PIC samples to be analyzed, we used 1/10 volume of PICs in this assay compared to the previous assays to reduce batch-to-batch variation in PIC activity. Therefore, the integration copies in this assay were expectedly lower than those measured in Fig. 2A. Nevertheless, the WT PICs showed markedly higher (~a log-fold) integration activity when compared to the CKI PICs, consistent with the results in Fig. 1 and 2. The addition of 0.5 µM CPSF6 enhanced the integration activity of WT PICs by ~2.5-fold, whereas 1.0 µM CPSF6 increased it by almost fourfold (Fig. 3B). However, further addition of CPSF6 (to 2.0 µM) failed to notably affect WT PIC activity, suggesting a saturating effect of the added CPSF6 between concentrations of 1 to 2 µM. In contrast, CKI cell-derived PICs showed a dose-dependent increase in integration activity up to the addition of 2 µM CPSF6 (Fig. 3C). Addition of 0.5 µM CPSF6 to the CKI PICs stimulated integration activity by ~2-fold, a level comparable to that of the control PICs. Moreover, a threefold stimulation in integration activity of CKI PICs was observed with 1.0 µM CPSF6 (Fig. 3D), which is slightly lower than that observed with the control PICs. By contrast to the WT condition, the addition of 2.0 µM CPSF6 resulted in a sevenfold higher integration activity of CKI PICs. Collectively, these biochemical studies provide additional evidence for a direct and stimulatory role of CPSF6 in PIC function.

### Disruption of CPSF6–CA interaction blocks HIV-1 DNA integration into the host genome

**Fig 4:**
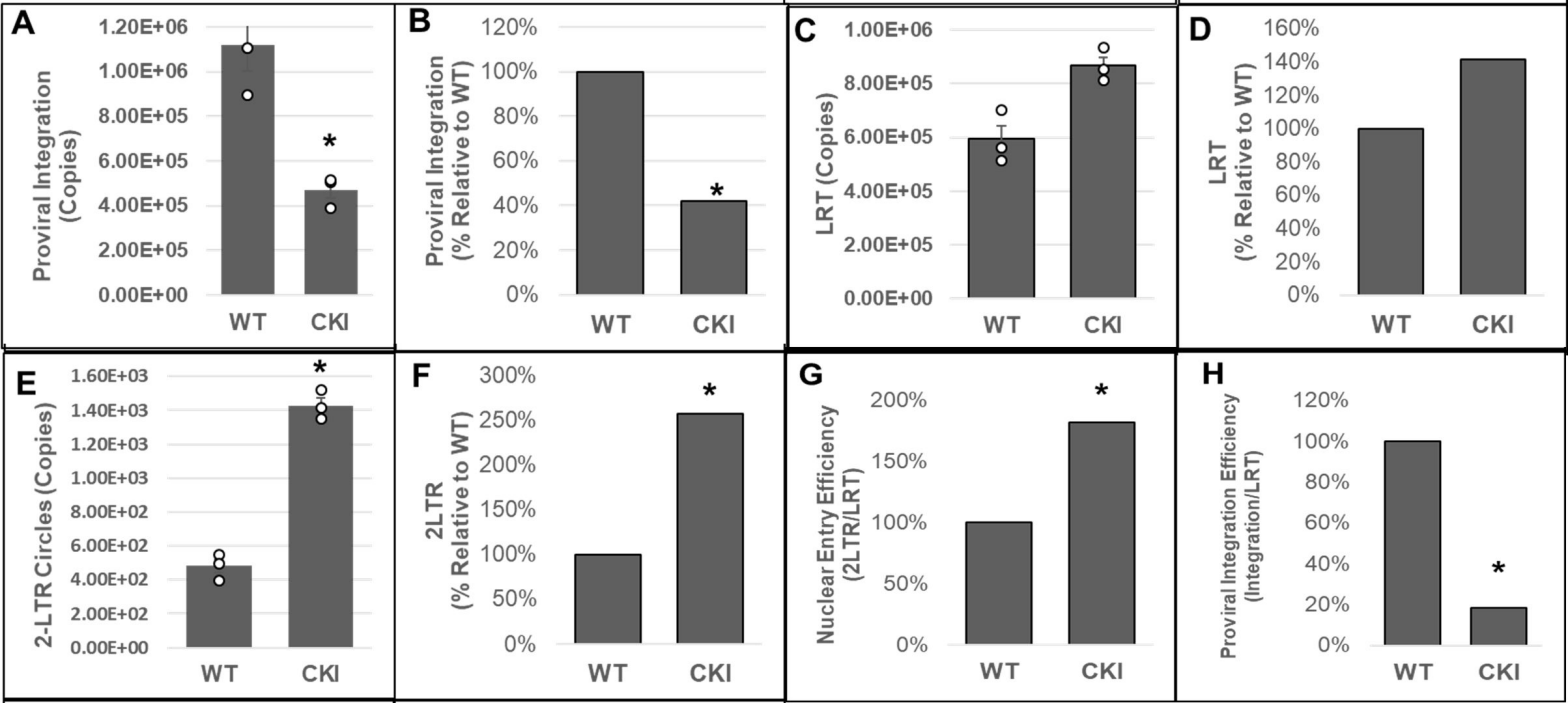
Disruption of the CPSF6–CA interaction blocks HIV-1 integration. (**A and B**) Integrated viral DNA was quantified by *Alu-gag* nested PCR using the total DNA extracted from infected CKI and WT control cells. Copy numbers were calculated from standard curves generated in parallel of known copy numbers (10^0^ to 10^8^) of an HIV-1 molecular clone during the second-round qPCR. (**A**) Copies of proviral integrants and (**B**) percentage of integrants relative to WT1 control cells. (**C and D**) Copies of late reverse transcription (LRT) products were measured by qPCR using a standard curve generated with known copy numbers of an HIV-1 molecular clone. (**C**) Copies of LRT and (**D**) percentage of LRT relative to the WT1 control cells. (**E and F**) 2-LTR circles were quantified from standard curves generated in parallel using known copy numbers of the p2LTR plasmid. (**E**) Copies of 2-LTR circles and (**F**) percentage of 2-LTR circles relative to the WT1 control cells. (**G**) A ratio of 2-LTR circles to LRT copies was calculated and plotted as relative percentage to the WT control cells. (**H**) A ratio of integration copies-to-LRT was calculated and plotted as relative percentage to the WT control cells. Data are mean values from three independent experiments, each conducted in triplicate with error bars representing the SEM. The *P*-values (*) represent statistical significance (*P* < 0.05) between the control and CKI cells. White circles present in graphs represent individual data points.

Our biochemical studies provided *in vitro* evidence for a critical role of CPSF6 in PIC-mediated viral DNA integration. The PIC is the subviral biochemical machinery that carries out viral DNA integration into the host genome (64). Therefore, to study the effect of CPSF6 on PIC function in an infected cell, we chronicled viral DNA synthesis and integration in the infected WT and CKI clonal cell lines. The WT and CKI SupT1 cells were individually inoculated with pseudotyped HIV-1 particles, and the total DNAs isolated from these cells were used to measure various forms of HIV-1 DNA, including integrated DNA detected via Alu-based nested qPCR (96, 105, 106). Results from these analyses showed a significant reduction (~75%) in the copies of integrated viral DNA in the CKI cells compared to the WT cells (Fig. 4A and B). These results suggest that the CPSF6–CA interaction promotes PIC-mediated viral DNA integration in infected cells.

HIV-1 integration is dependent on the prior steps of reverse transcription and nuclear import (64). Notably, HIV-1 CA is known to regulate reverse transcription and post-reverse transcription steps of infection (16, 52, 55, 83), and the CPSF6–CA interaction is implicated in nuclear entry and post-nuclear entry steps of infection (75, 78, 81, 83, 107–109). Therefore, we investigated whether the integration defect in the CKI cells was a consequence of a prior block at the reverse transcription and/or nuclear entry steps. Copies of late reverse transcription (LRT) products were measured by qPCR using the total DNA from the cells. These measurements revealed that viral DNA levels were not reduced in the CKI cells when compared to the WT cells; rather, they were slightly elevated (~40%) (Fig. 4C and D). These results suggested that the disruption of CPSF6–CA interaction does not reduce reverse transcription, and the integration block is likely manifested at a post-reverse transcription step. Therefore, we next quantified the levels of 2-LTR circles, a commonly used surrogate marker of nuclear import of retroviral DNA, that are generated in the nucleus of an infected cell by non-homologous end-joining (110–114). Copy numbers of 2-LTR circles in infected CKI cells as well as the WT control cells were calculated by extrapolating qPCR data to a standard curve generated using a plasmid containing the HIV-1 2-LTR junction sequence. Unexpectedly, we observed an increase (~2.5-fold) in the number of 2-LTR circles in the CKI clones relative to the WT cells (Fig. 4E and F). Moreover, calculation of the efficiency of nuclear entry (ratio of late RT copies to 2-LTR copies) revealed that the nuclear entry of HIV-1 DNA was not reduced in CKI cells, but was seemingly stimulated (Fig. 4G), an observation similar to the reverse transcription in these cells. Finally, the calculation of the efficiency of integration (ratio of integration copies to late RT copies) revealed a reduction (~80%) in integration efficiency in the CKI cells (Fig. 4H). These results provide further support that CPSF6 contributes to PIC-mediated viral DNA integration in infected cells.

### Disruption of CPSF6–CA interaction minimally affects integration of HIV-1 CA mutant viruses deficient in CPSF6 binding

**Fig 5:**
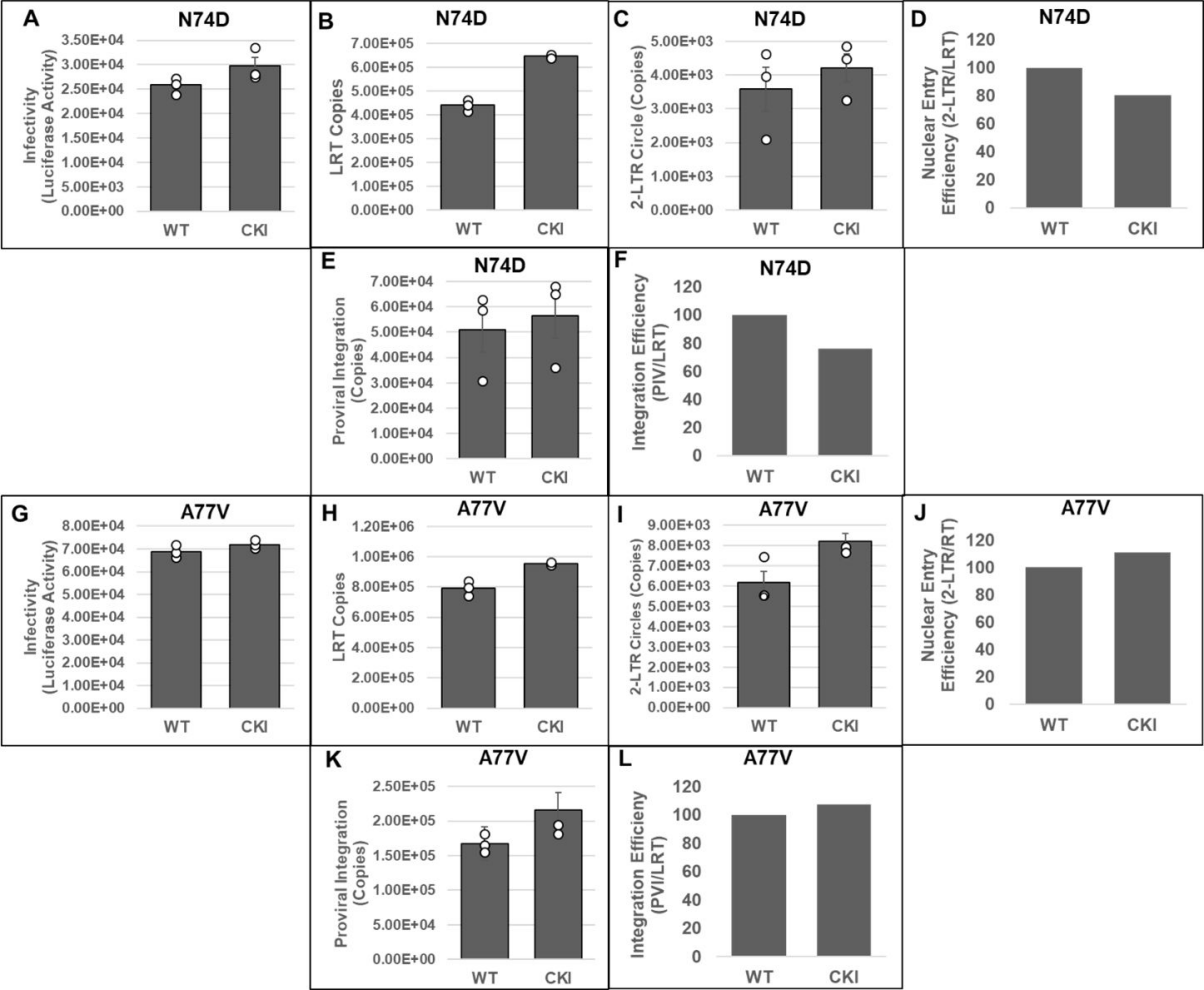
Measurement of early infection steps of HIV-1 CA mutant viruses disrupted for CPSF6 binding. (**A and G**) Infectivity measurements. WT and CKI clones inoculated with WT CA or mutant CA HIV-1 particles (N74D, panels A–F, and A77V, panels G–L) were cultured for 24 h, and luciferase activity was measured in the cellular lysates. (**B and H**) Quantification of LRT copies as a measure of reverse transcription. (**C and I**) Quantification of 2-LTR circle copies as a measure of nuclear entry. (**D and J**) Normalized levels of nuclear entry. (**E and K**) Integrated viral DNA copies were quantified by *Alu-gag* nested PCR assay as described in Fig. 1. (**F and L**) A ratio of the copies of proviral integration to LRT copies as a measure of integration efficiency. Data are plotted as relative percentage to the WT control cells. Data are mean values from at least three independent experiments, each conducted in triplicate with error bars representing the SEM. White circles present in graphs represent individual data points.

To solidify CPSF6’s role in HIV-1 integration, we probed the early steps of infection of HIV-1 CA mutant viruses N74D and A77V, which are deficient for CPSF6 binding (76, 77). We inoculated CKI and control cells with pseudotyped luciferase reporter N74D (Fig. 5A) or A77V (Fig. 5G) HIV-1 particles. At 24 hpi, luciferase activity was measured in the cellular lysates as an indicator of infectivity. As expected, the infectivity levels of the mutant viruses in CKI cells were comparable to those in control cells, confirming that the infectivity of these mutants is not dependent on CPSF6 binding. Then, we measured the levels of LRT, 2-LTR circles, and viral DNA integration in these cells by qPCR. These measurements indicated that the copies of LRT synthesized by N74D (Fig. 5B) or A77V (Fig. 5H) were not reduced in CKI cells when compared to the control cells. Moreover, there was a slight increase in the LRT levels of these CA mutants in the CKI cells, an effect similar to that observed for WT HIV-1 in CKI cells (Fig. 4C and D). We also observed that the number of 2-LTR circles formed by the mutant viruses in the infected CKI cells was comparable to those formed in the control cells (Fig. 5C and I). Calculation of the nuclear import efficiency indicated a slight but non-significant decrease in nuclear import of the N74D virus in the CKI cells when compared to the control cells (Fig. 5D). However, no such reduction in nuclear import efficiency was detected with the A77V virus in the CKI cells (Fig. 5J). Finally, estimation of the copies of viral DNA integration revealed that integration of N74D (Fig. 5E) or A77V (Fig. 5K) virus was not reduced in the CKI cells when compared to the control cells. Estimation of integration efficiency indicated a slight decrease in N74D integration in CKI cells (Fig. 5F) but no change in the case of the A77V mutant virus (Fig. 5L). The level of decrease in N74D integration (~20%) matched the reduction in nuclear import (~20%) in the CKI cells, suggesting that the integration defect was most likely due to the reduction in nuclear import. Together, these results suggest that HIV-1 CA mutants incapable of binding CPSF6 are not blocked for viral DNA integration in CKI cells, thus providing additional evidence supporting an important role for CPSF6 in HIV-1 integration.

### Disruption of the CPSF6–CA interaction retargets HIV-1 DNA integration away from SPADs and into LADs

**Fig 6:**
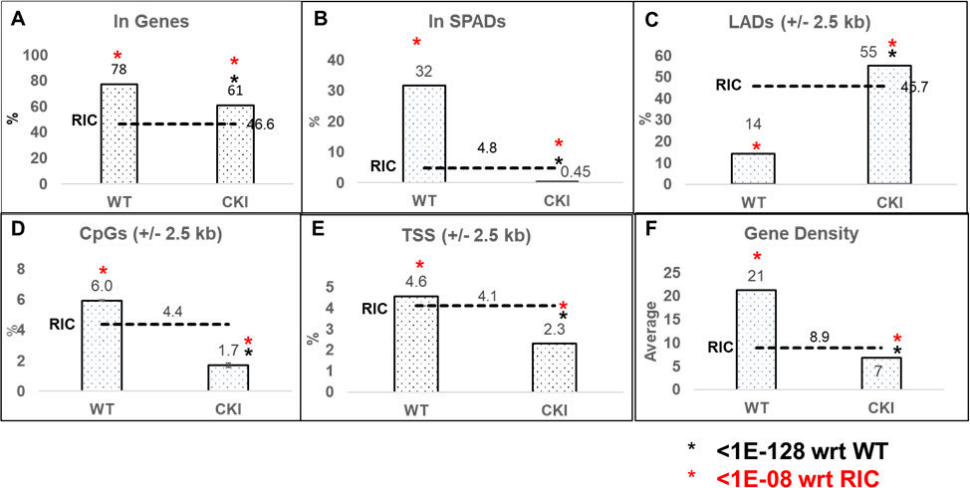
Disruption of CPSF6–CA Interaction retargets HIV-1 DNA away from SPADs and into LADs. Integration into (**A**) genes and (**B**) SPADs was scored as within these genomic coordinates. For (**C**) LADs, (**D**) CpG islands, and (**E**) TSSs, sites were mapped within ±2.5 kb windows (5 kb surrounding these coordinates). (**F**) Gene density within the 500 kb surrounding integration sites. Gene density was assessed as the number of genes per Mb. RICs were generated by shearing hg19 *in silico* using the restriction enzyme sites used to generate the wet bench samples and then mapping the resultant fragments with respect to the aforementioned genomic annotations. The *P*-values (*) represent statistical significance with respect to (wrt) WT cells (black asterisks; <10^−128^) and matched RIC values (red asterisks; <10^−8^).

The CPSF6–CA interaction is important for viral cores to move distal from the nuclear rim, the consequences of which help to target viral DNA integration into gene-rich SPADs (75, 78, 82, 83, 90, 115). Accordingly, CPSF6 depletion or disruption of CPSF6–CA binding misdirects PIC-mediated integration into LADs, which are proximal to the nuclear envelope. Our results indicated that CPSF6 is critical for HIV-1 DNA integration, both *in vitro* (Fig. 1 to 3) and in infected cells (Fig. 4). Therefore, to further understand the role of CPSF6 in PIC function, we mapped proviral integration sites in CKI and control cells. Genomic DNAs isolated from infected cells were sheared and ligated to asymmetric linkers, and the resulting DNAs were purified. Then, LTR-linker sequences were amplified via a semi-nested PCR assay and were analyzed by Illumina sequencing (116, 117). Unique sites of HIV-1 integration were then mapped with respect to several genomic features, including genes, SPADs, LADs, CpG islands, TSSs, and gene-dense regions, as well as computationally generated RIC values (Fig. 6). In control cells, as expected, HIV-1 integration into genes was significantly enriched when compared to the RIC value (Fig. 6A). However, in the CKI cells, there was a significant reduction in integration into genes (Fig. 6A). Notably, the integration sites within the CKI cells were significantly retargeted away from SPADs, relative to the integration sites in the control cells (Fig. 6B). For example, SPAD-proximal integration in WT cells was approximately 35%, whereas in the CKI cells, it was significantly reduced to ~0.5% (Fig. 6B), a value significantly lower than the 4.8% RIC value for SPAD-proximal integration. Expectedly, there was a concurrent and significant increase in integration into LADs in the CKI cells (~55%), whereas, not surprisingly, integration into LADs was significantly lower in the WT1 cells (Fig. 6C). Furthermore, while 1.7% and 2.3% integrations were within 2.5 kb of CpG island and TSS, respectively, in CKI cells, 6% and 4.6% integrations were within the CpG island and TSS, respectively, in control cells (Fig. 6D and E). Finally, integrations into gene-dense regions of chromatin were significantly reduced in CKI cells, from about 21 genes/Mb in WT cells to 7 genes/Mb, which was significantly lower than the 8.9 genes/Mb RIC value (Fig. 6F). These integration site analyses indicate that disrupting the CPSF6–CA interaction profoundly redirects the PIC away from SPADs toward the low-gene-dense regions of LADs near the nuclear envelope for viral DNA integration.

### Effect of CPSF6–CA interaction on HIV-1 infectivity is context dependent

**Fig 7:**
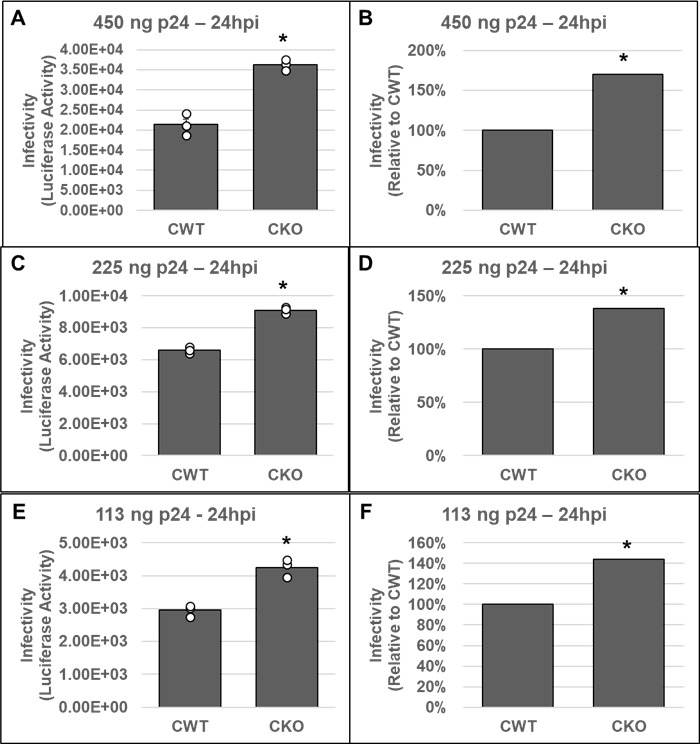
Effects of CPSF6 depletion on HIV-1 infectivity. CKO and control HEK293T (CWT) cell lines were inoculated with three different concentrations of pseudotyped HIV-1.Luc virus as described in Materials and Methods; the total infection time course was 24 h. Cell pellets were then collected and subjected to infectivity evaluation by measurement of luciferase activity. Infectivity is displayed as both luciferase activity (**A, C, E**) and as a percentage relative to the luciferase activity of the corresponding CWT control (**B, D, F**). Three virus concentrations included (**A, B**) 450 ng p24, (**C, D**) 225 ng p24, and (**E, F**) 113 ng p24. Data are representative of three independent experiments, each conducted in triplicate with error bars representing the SEM. The *P*-values (*) represent statistical significance (*P* < 0.05) in CWT and CKO cells. White circles present in graphs represent individual data points.

Our *in vitro* and cellular studies of HIV-1 integration strongly suggested that CPSF6 promotes HIV-1 PIC function. To probe how the effects of CPSF6 on PIC function influenced HIV-1 infectivity, we carried out single-round infection studies in both CKO and CKI cells. First, we inoculated CKO and CWT HEK293T cells with three different amounts of pseudotyped luciferase reporter HIV-1 particles. At 24 or 48 hpi, luciferase activity was measured in the cellular lysates as an indicator of virus infectivity. Results from these assays showed that, compared to the CWT cells, HIV-1 infection was stimulated in CKO cells at both 24 hpi (Fig. 7) and 48 hpi (Fig. S3). The increased infectivity in the CKO cells was sustained at all three concentrations of virus used for inoculation (Fig. 7; Fig. S3). These infectivity results are consistent with previously reported data from studies using these cells (82).

**Fig 8:**
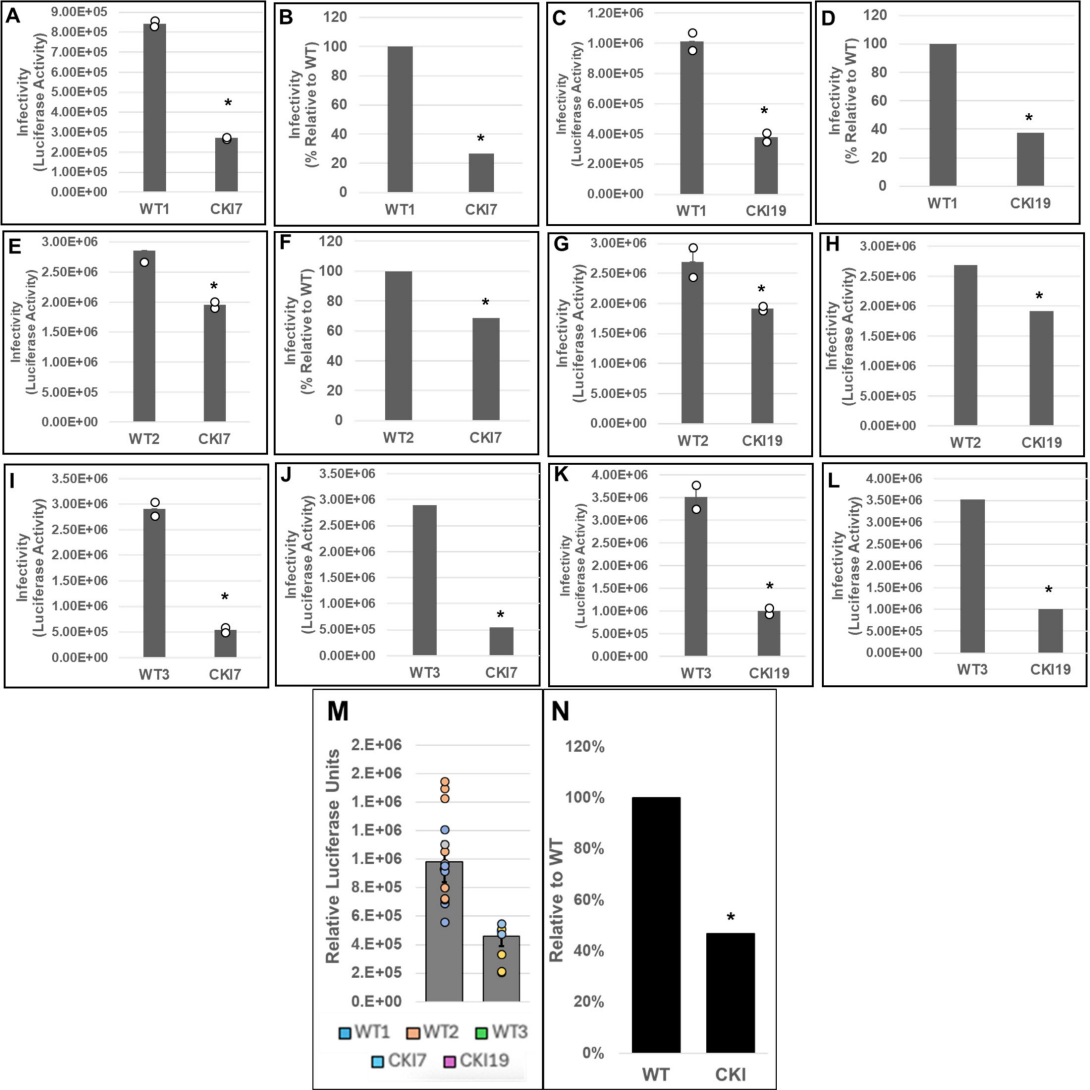
Effects of disrupting the CPSF6–CA interaction on HIV-1 infection at 24 hpi. WT cell lines WT1 (**A–D**), WT2 (**E–H**), and WT3 (**I–L**) and CKI SupT1 cell lines CKI7 (A, B; E, F; and I, J) and CKI19 (C, D; G, H; and K, L) were spinoculated with 450 ng p24 of pseudotyped HIV-1.Luc reporter particles. The total infection time course was 24 h, and luciferase activity was measured in the cellular lysates as an indicator of infectivity (panels A, C, E, G, I, and K). Infectivity data were also plotted as percent infectivity relative to respective control clones (panels B, D, F, H, J, and L). Data shown are mean values from three independent experiments, each conducted in triplicate. (**M**) Infectivity values sorted by editing treatment (CPSF6 non-edited vs edited). Colored circles represent individual data points from corresponding cell line. (**N**) Aggregate infectivity as a percent relative to WT. Error bars represent the SEM, and the *P*-values (*) represent statistical significance (*P* < 0.05) between the control and CKI cells.

**Fig 9:**
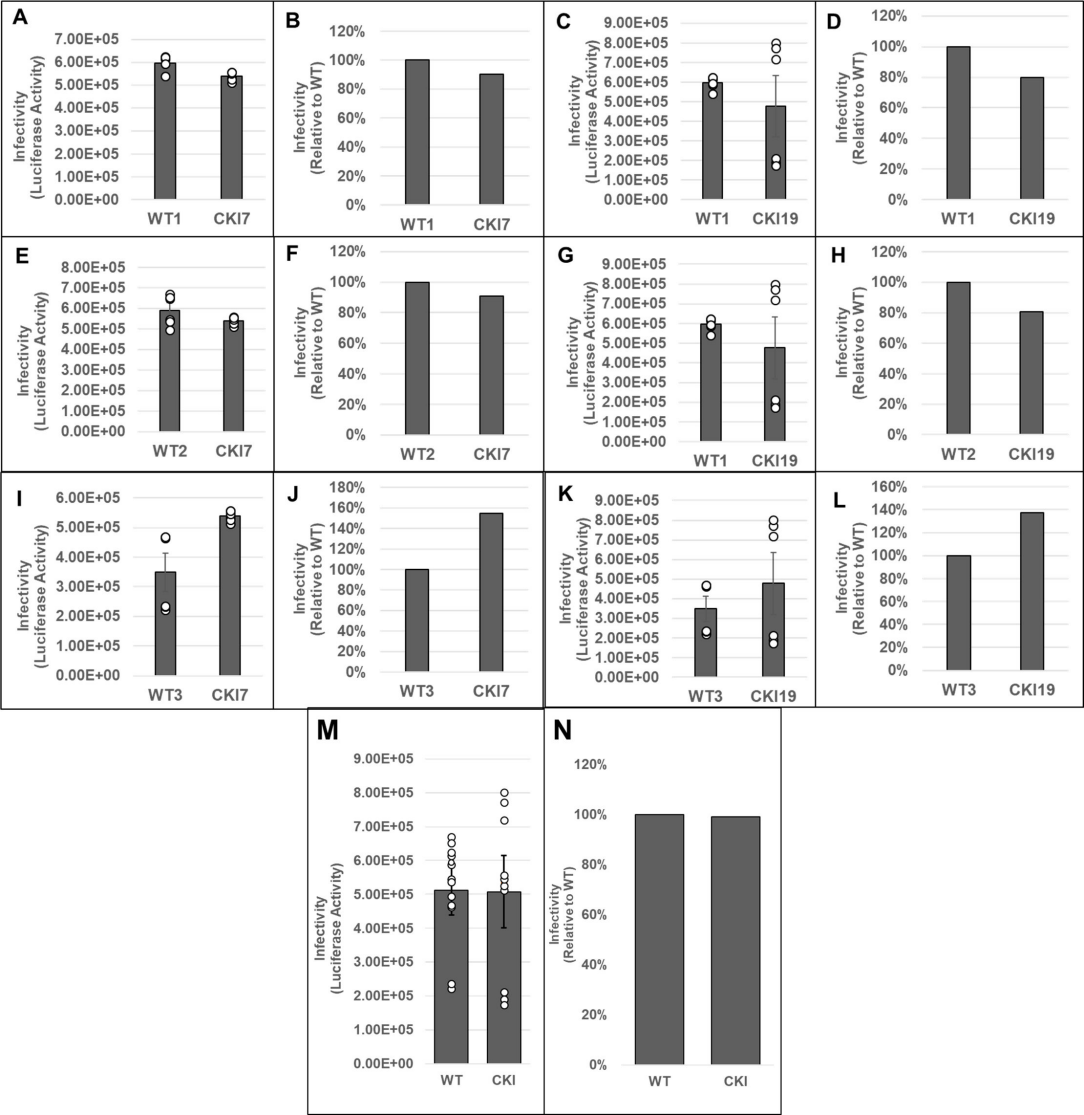
Effects of disrupting the CPSF6–CA interaction on HIV-1 infection at 48 hpi. These data are identical to Fig. 8, except that the infection time course was for 48 h. (A, C, E, G, I, and K) luciferase activity; (B, D, F, H, J, and L) percent infectivity relative to respective control clones. (**M**) Infectivity values sorted by editing treatment (CPSF6 non-edited vs edited). Colored circles represent individual data points from corresponding cell line. (**N**) Aggregate infectivity as a percent relative to WT. Data shown are mean values from three independent experiments, each conducted in triplicate. Error bars represent the SEM, and the *P*-values (*) represent statistical significance (*P* < 0.05) between the control and CKI cells.

Next, three parental (WT control) and two CKI SupT1 clones were individually inoculated with pseudotyped luciferase reporter HIV-1 particles, and virus infectivity was measured at 24 or 48 hpi. These measurements showed that the WT control cells supported higher virus infectivity when compared to the CKI clones at 24 hpi (Fig. S4A). Normalization of the luciferase activity to the total protein levels or to the viability of the respective infected cells minimally changed the level of reduction in virus infectivity in the CKI clones (Fig. S4B and C). To comprehensively probe the virus infectivity levels in the CKI clones, we performed pairwise matched infections of WT versus CKI cell lines (Fig. 8). Results from these analyses showed a reduction in viral infectivity in both CKI cell lines at 24 hpi, when compared across control cell lines. Particularly, when compared to WT1 control cells, an estimated 70% and 60% decrease in infectivity was observed in CKI7 (Fig. 8A and B) and in CKI19 (Fig. 8C and D) cells, respectively. However, when compared to WT2 cells, an estimated 30% decrease in infectivity was observed for both CKI7 (Fig. 8E and F) and CKI19 (Fig. 8G and H) cells. Conversely, when compared to WT3 cells, an estimated 80% and 70% decrease in infectivity was observed for CKI7 (Fig. 8I and J) and CKI19 (Fig. 8K and L) cells, respectively. Considering the large clonal-based variation in infectivity levels in these recoded SupT1 cells, we compared the aggregate infectivity data of all WT and CKI cell lines (Fig. 8M and N). These results revealed that HIV-1 infectivity was significantly reduced by ~50% across CKI cells compared to the WT cells. Notably, by 48 hpi, the CKI infection defects noted at 24 hpi had dissipated, both at the level of cell type-paired match infections (Fig. 9A through L) and aggregate cell infection data (Fig. 9M and N). A minimal change in HIV-1 infectivity at 48 hpi was previously reported in CPSF6-depleted Jurkat cells in single-round infection assays (90).

## DISCUSSION

HIV-1 infection is dependent on the integration of PIC-mediated viral DNA into gene-dense regions of the host genome (64, 118). Particularly, SPADs and speckle-associated chromatin, which lie in close proximity to nuclear speckles, are highly favored targets for HIV-1 integration (115). A growing body of literature supports that CPSF6 is responsible for targeting the HIV-1 PIC to nuclear speckles for subsequent IN-mediated integration into SPADs (75, 78, 82, 83, 90, 115). However, several key questions underlying the mechanism by which CPSF6 promotes integration targeting remain unanswered. These include (i) does the engagement of CPSF6 with HIV-1 CA at a specific location (cytoplasm, NPC, nucleoplasm, or speckles) dictate PIC targeting, (ii) are there other viral or host factors that cooperate with CPSF6 for integration targeting, (iii) does CPSF6 remain bound to the PIC-associated CA until integration, and (iv) importantly, what role does CPSF6 play in PIC function? In this study, we probed the effects of CPSF6 on PIC-mediated viral DNA integration *in vitro* and in infected cells.

To test a direct role of CPSF6 in HIV-1 integration, we first studied the PIC—the sub-viral biochemical complex that carries out viral DNA integration in an infected cell (64, 65). Due to technical challenges associated with reproducible extraction of PICs from isolated nuclei, we studied PICs extracted from the cytoplasm of infected cells. We found that the PICs from CKO cells exhibited significantly lower integration activity, and calculation of specific PIC activity demonstrated that the lower activity of CKO PICs was not due to lower number of PICs formed in these cells (Fig. 1). Similarly, the integration activity of PICs extracted from CKI SupT1 cells, recoded to disrupt the CPSF6–CA interaction, exhibited significantly lower integration activity when compared to WT SupT1 cell PICs (Fig. 2). Interestingly, the relative reduction in the integration activity of CKI SupT1 PICs was more substantial than the relative reduction in the integration activity of CKO cell PICs. Although consistent reduction in integration activity was detected in PICs extracted from CKO and CKI cells, the variability in integration activity reduction could accordingly be due to cell-type dependency. Therefore, to solidify a direct role for CPSF6 in HIV-1 integration, we carried out *in vitro* integration activity measurements of PICs in the presence of purified CPSF6 protein (Fig. 3). Addition of CPSF6 protein stimulated the activity of PICs extracted from both CKI and control cells (Fig. 3). However, the extent of increase in integration activity in the presence of CPSF6 was higher for the CKI PICs when compared to the control PICs. Considering the significantly lower integration activity of CKI PICs, supplementation with CPSF6 most likely helps in the partial restoration of the integration activity of these PICs. Indeed, supplementation with 2.0 µM CPSF6 led to a ~7-fold increase in the integration activity of CKI PICs. Conversely, the lack of stimulation in the activity of control PICs with 2.0 µM CPSF6 suggested a possible saturating effect of CPSF6 on PIC function. These biochemical studies suggest that PIC integration activity is stimulated by CPSF6, and optimal PIC activity depends on the CPSF6–CA interaction.

Our biochemical studies provided *in vitro* evidence for a direct and functional role of CPSF6 in viral DNA integration by the PIC. Next, we sought to validate the relevance of this observation in HIV-1-infected target cells. For this, we probed CPSF6’s role in HIV-1 integration into the host genome. Our results demonstrated that HIV-1 integration was significantly reduced in CKI cells when compared to WT control cells (Fig. 4A and B). HIV-1 CA mutants incapable of binding to CPSF6 showed no such integration defect in CKI cells when compared to the control cells (Fig. 5), thus confirming that CPSF6’s positive effect on integration is dependent on its binding to the viral capsid. However, HIV-1 integration is dependent on both viral DNA synthesis and viral DNA import into the nucleus (64). Either of these steps could be influenced by CPSF6–CA binding, since CA regulates both reverse transcription and post-reverse transcription steps (45, 46, 83, 84, 118–121) and the CPSF6–CA interaction affects nuclear entry and post-entry steps of HIV-1 infection (109, 115, 122). Therefore, we probed whether CPSF6’s effect on HIV-1 integration was dependent on the preceding steps of viral DNA synthesis and nuclear entry. Notably, viral DNA synthesis was stimulated rather than inhibited in CKI cells (Fig. 4C and D), implying that engagement of CPSF6 with the HIV-1 capsid may negatively impact viral DNA synthesis in T cells. Previous analyses of cytoplasmically mislocalized C-terminal truncation CPSF6 variants reported significant reductions of viral DNA synthesis (75, 79, 123). Moreover, the higher level of viral DNA synthesis in CKI cells translated to increased HIV-1 nuclear entry (Fig. 4E and F). This was surprising, as CPSF6 depletion was previously reported to yield marginal reductions in HIV-1 nuclear import in certain cell types (78, 107). Nevertheless, we predict that lack of CPSF6 binding may allow prolonged engagement of CypA with the capsid, since CypA is known to prevent CPSF6-358-mediated restriction of the HIV-1 capsid (84). Thus, CypA-mediated stabilization of the capsid could plausibly provide a microenvironment suitable for more efficient reverse transcription in CKI cells. In this scenario, higher viral DNA synthesis would generate a higher number of 2-LTR circles because only a very small percentage of viral DNA is integrated into the host genome (124). However, detailed kinetic studies of viral DNA synthesis, 2-LTR circle formation, and viral DNA integration in cells depleted of either CypA or CPSF6 or both are required to validate this speculation. Additionally, whether the stimulation of nuclear entry in the CKI cells was due to the utilization of alternative nuclear import pathways (55, 68, 92) requires further investigation.

Our studies established that CPSF6 promotes HIV-1 PIC activity, and disruption of CPSF6–CA binding reduces viral DNA integration into the host genome. It is well established that PIC-associated viral DNA is preferentially integrated into active chromatin including highly transcribed genes, gene-dense regions, activating epigenetic marks, and SPADs (83, 118, 125). By contrast, HIV-1 integration is disfavored in heterochromatin regions containing repressive epigenetic marks and LADs (81, 125). One of the primary functions of CPSF6 is to direct the PIC away from the LADs located near the nuclear periphery to promote selective integration into SPADs (75, 78, 82, 83, 90, 115). So, when CPSF6 is depleted or CPSF6 cannot bind to capsid, the PIC remains closer to the nuclear lamina leading to integration into LADs (54, 78, 81, 107, 126). Therefore, we asked if HIV-1 integration targeting was altered in CKI cells due to the loss of the CPSF6–CA interaction. To test this, we mapped HIV-1 integration into genomic features, including genes, SPADs, LADs, CpG islands, TSSs, and gene-dense regions (Fig. 6). Predictably, HIV-1 integration into genes, gene-dense regions, and SPADs was significantly enriched in the control cells. In contrast, there was a significant reduction in integration into genes, gene-dense regions, and SPADs in the CKI cells. Concurrently, the LAD regions served as a major target for viral DNA integration in the CKI cells. This profound integration retargeting is a consequence of the lack of CPSF6 binding to the HIV-1 capsid.

Our *in vitro* and cell-based assays demonstrated that CPSF6 is important for HIV-1 DNA integration. Since viral DNA integration is required for productive infection, we carried out single-round infection studies in both CKO and CKI cells. As previously reported (82), higher HIV-1 infection was observed in HEK293T CKO cells at both 24 hpi (Fig. 7) and 48 hpi (Fig. S3). The effects of CPSF6 on HIV-1 infection seem to be cell-type dependent. For example, HIV-1 infection has been reported to be marginally enhanced in CKO HEK293T cells and certain CPSF6-depleted cell lines in single-round infection assays (67, 82, 127, 128). In contrast, multi-round HIV-1 replication was reduced in CPSF6-depleted primary macrophages (79). Similarly, depletion of CPSF6 in primary, resting CD4+ T cells reduced HIV-1 infection in single-round infection assays (129). Nevertheless, these prior cellular models were instrumental in defining CPSF6’s role during HIV-1 nuclear entry, nuclear trafficking, and integration targeting. However, these cell line models were limited in probing a direct role of CPSF6 in PIC function since CPSF6 regulates multiple steps before integration, and each of these steps could affect PIC function. Additionally, prior CKO cell lines of HEK293T and HeLa cell origin were sub-optimal to address whether CPSF6’s effect on PIC function was a direct consequence of CA binding or perhaps due to an indirect effect of alterations in cellular physiology associated with depletion of a key cellular protein. To circumvent such limitations, we recoded the critical FG motif in CPSF6 in SupT1 cells (Fig. S1 and S2). Single-round infection assays showed significant reduction in viral infectivity in two different CKI cell lines at 24 hpi (Fig. 8). However, by 48 hpi, this infectivity defect had dissipated (Fig. 9). Collectively, these results suggest that the detrimental effect of disrupting the CPSF6–CA interaction on HIV-1 infection is short-lived, which is consistent with the notion that CPSF6’s effect on HIV-1 infection is both context and cell-type dependent.

In summary, our results described in this report, using a combination of genetic, virology, molecular biology, and biochemical approaches, indicate a direct role for CPSF6 in HIV-1 PIC function both *in vitro* and in infected cells. Our studies support the prediction that CPSF6 promotes viral DNA integration by binding to PIC-associated CA (54, 130, 131). There is evidence that CypA protects the capsid from restriction by cytosolic CPSF6 (132) and promotes PIC activity (133). As the core-associated PIC is trafficked through the nucleoplasm toward nuclear speckles, CPSF6 and CypA could cooperatively regulate the level of PIC-associated CA. When CPSF6 binding is disrupted, CypA could continue to be associated with the capsid, leading to insufficient removal of PIC-associated CA and consequently impaired integration. This prediction is supported by our recent report that CypA prevents integration of certain HIV-1 CA mutants that escape the inhibitory effect of CD8+ cytotoxic T lymphocytes (CTLs) (100). However, CypA depletion removes the integration block of these CTL-escape mutants, thus suggesting CypA can be inhibitory to PIC function under certain conditions. The capsids of these CTL-escape CA mutants retain the ability to bind to CPSF6, implying that CPSF6 binding may negatively affect the removal of PIC-associated CA in the mutant viruses. Additionally, the retention of excess CA may reduce PIC function by interfering with the recently reported role of CPSF6 in liquid-liquid phase separation (75, 134–136). Future studies are required to pinpoint the mechanism by which CPSF6 regulates HIV-1 PIC function.

## Data Availability

All the data generated in this study are included in the article as either main figures or supplemental figures.

## SUPPLEMENTAL MATERIAL

Supplemental material jvi.00490-25-s0001.pdf Figures S1 to S4.

ASM does not own the copyrights to Supplemental Material that may be linked to, or accessed through, an article. The authors have granted ASM a non-exclusive, world-wide license to publish the Supplemental Material files. Please contact the corresponding author directly for reuse.
