# Supplementary material for: CPSF6 promotes HIV-1 preintegration complex function
Source: J Virol. 2025 Apr 9;99(5):e00490-25. doi: 10.1128/jvi.00490-25 (PMC12090733; doi:10.1128/jvi.00490-25)
Supplement: Supplemental material — Figures S1 to S4. [file jvi.00490-25-s0001.pdf]

Supplementary Data

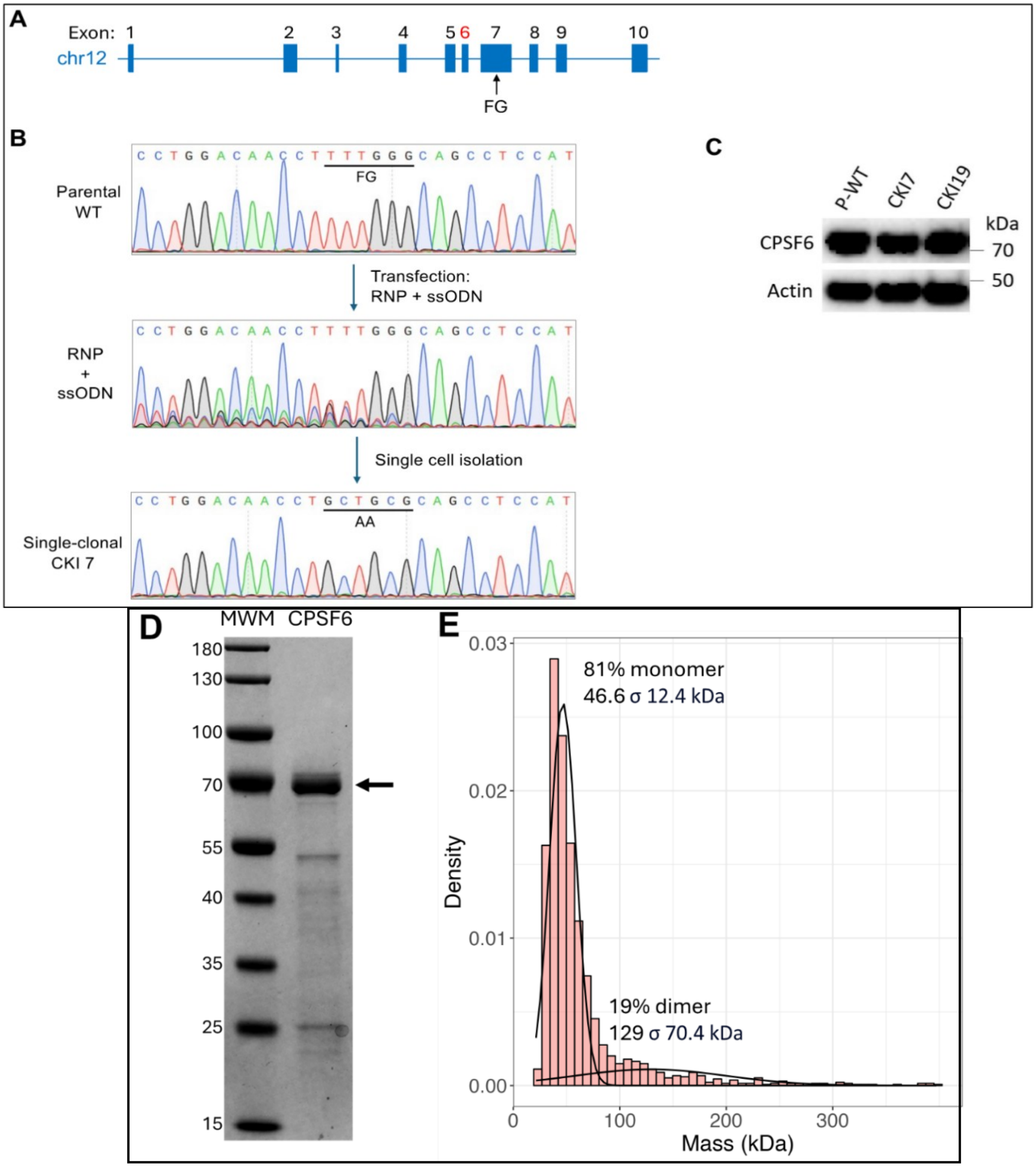

Fig. 1: (A-C) Generation of CKI SupT1 cells by CRISPR-Cas9. (A) Schematic of human *CPSF6*; the FG motif is encoded by DNA sequences within exon 7. Exon 6, in red, is most usually removed via alternative splicing. **(B)** Sanger sequencing results of PCR amplicons derived from parental SupT1 cells (upper), transfected cells prior to limited dilution cloning (middle), and the clonally expanded CKI17 cell line. Targeted nucleotides are underlined. **(C)** Immunoblot of CPSF6 expression levels in parental SupT1 cells versus clonally-expanded CKI7 and CKI19 cell lines. **(D-E) Characterization of recombinant CPSF6 protein purified from *E. coli* bacteria. (D)** Coomassie blue-stained image of the purified protein (2 µg), which is indicated by the arrow. Quantification using ImageJ software indicated that the protein was 79% pure. Numbers to the left are the kDa's of the shown mass standards. **(E)** Multimeric state (10 nM total protein) as assessed by mass photometry. The theoretical mass of CPSF6 is 59.2 kDa.

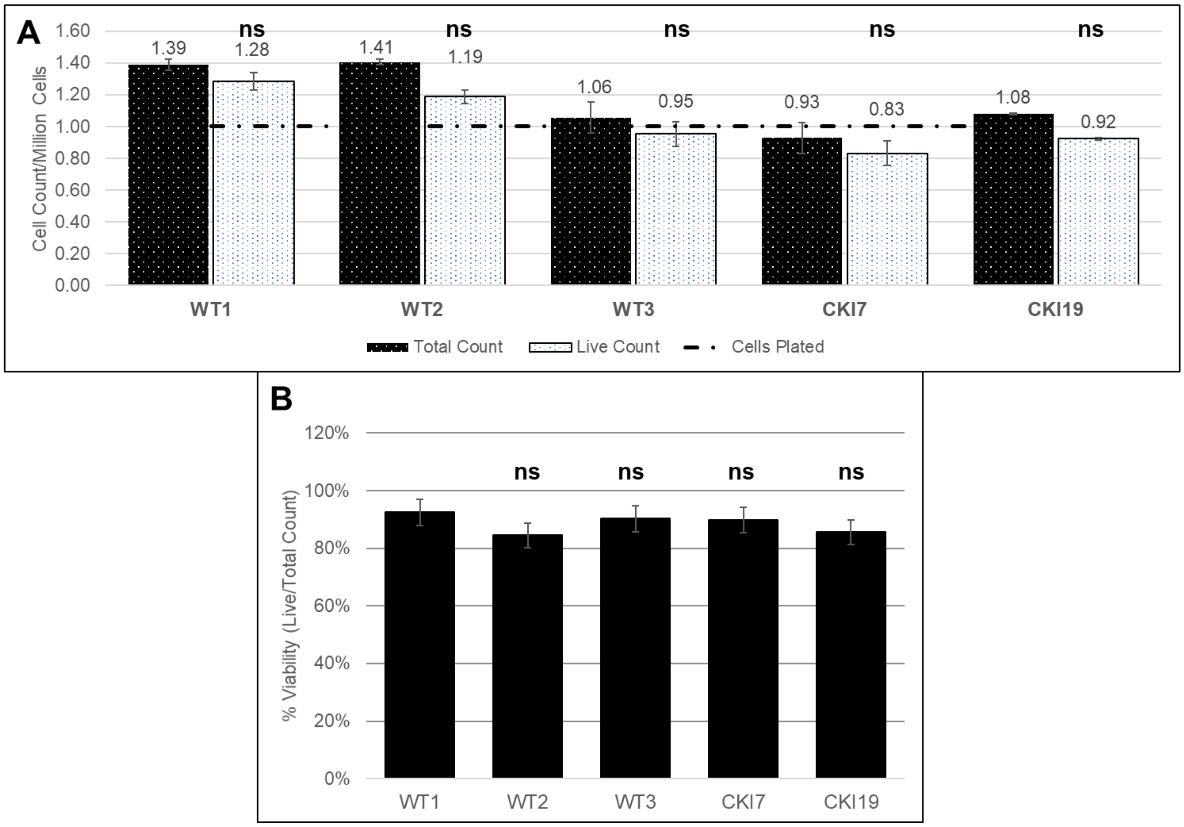

Fig. 2: Viability of CKI and WT SupT1 cells. SupT1 cells were compared amongst each other in terms of live and total cell count to assess relative cell viability. Cells (10^6) were counted prior to seeding in culture plate for 24 h. Following incubation, cells were once again counted and assessed for total cell count, live cell count **(A)** and cell viability **(B)**. Dashed line represents the number of cells seeded prior to seeding. Black patterned bar is representative of an average of 3 total cell counts whereas the white patterned bar is representative of 3 live cell counts. Both values were normalized to 10^6 cells. **(B)** Cell viability (live cell count/total cell count) as a percentage of total cells counted. Error bars are representative of SEM. The p-values (*) represents statistical significance ($p < 0.05$) compared to WT1. NS represents non-significant difference as compared to WT1.

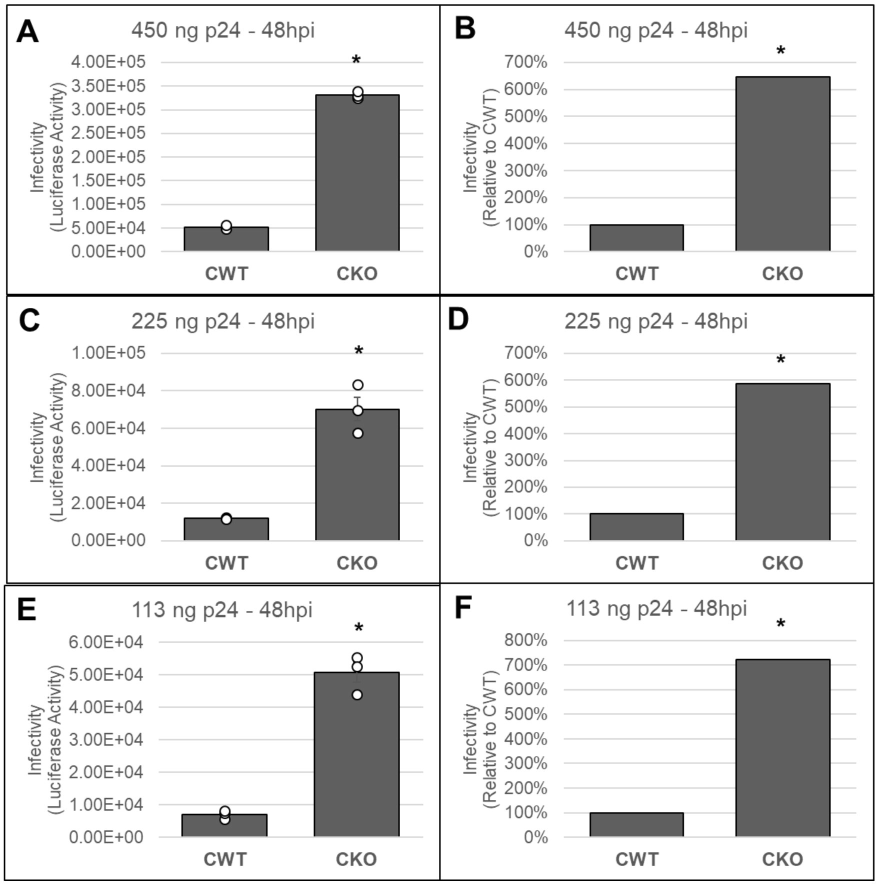

Fig. 3. Effects of CPSF6 depletion on HIV-1 infectivity at 48 hpi. HEK293T cells (10^6) were seeded in 6-well plates for 24 h prior to virus inoculation. Following incubation, cell lysates were collected and were processed for assessment of luciferase activity- a surrogate for infectivity. **(A, C, E)** Infectivity at 3 concentrations by p24 content of the viral particle: 450 ng **(A)** 225 ng **(B)** 113 ng **(C).** (**B, D, F)** Infectivity measurements as a percentage relative to CWT. All experiments were conducted with three independent replicates with 3 technical replicates during the luciferase measurement. **(A, C, E**) Data displayed with error bars representing SEM and circles representing replicate data points. The p-values (*) represents statistical significance (p< 0.05) compared to CWT.

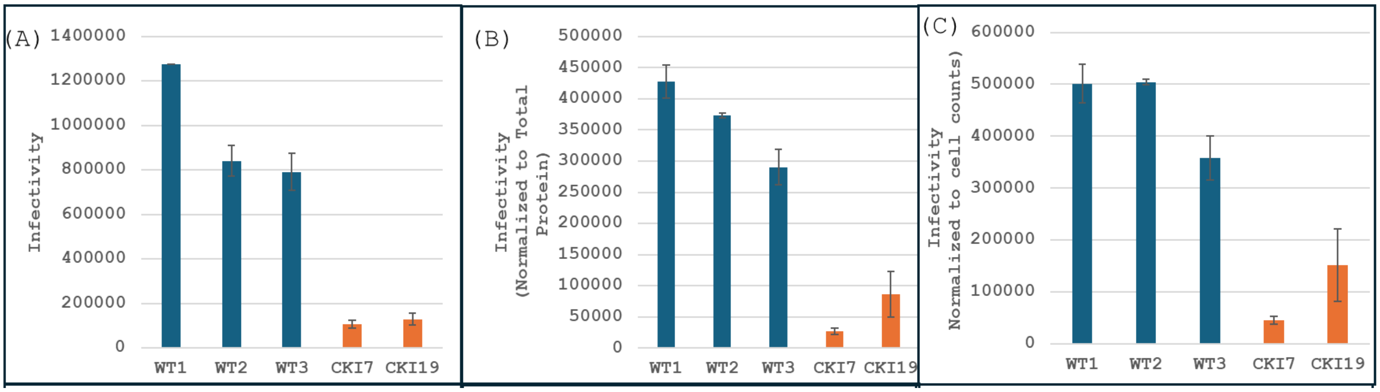

Fig. 4: Infectivity in CKI and WT control SupT1 cells. Cells were seeded in 6-well culture plates and inoculated with pseudotyped HIV-1.Luc reporter virus. The entire infection time course spanned 24 h. Subsequently, cell lysates were aliquoted for further processing for total protein estimation (via BCA assay) and luciferase as a surrogate for infectivity. Data shown are representative of mean values from three independent experiments. **(A)** Infectivity as represented strictly by luciferase activity. **(B, C)** Infectivity values normalized to either protein or total cell count respectively. Error bars represent SEM.
